# The Inorganic Nutrient Regime and the *mre* Genes Regulate Cell and Filament Size and Morphology in the Phototrophic Multicellular Bacterium *Anabaena*

**DOI:** 10.1128/mSphere.00747-20

**Published:** 2020-10-28

**Authors:** Cristina Velázquez-Suárez, Ignacio Luque, Antonia Herrero

**Affiliations:** a Instituto de Bioquímica Vegetal y Fotosíntesis, CSIC and Universidad de Sevilla, Seville, Spain; University of Wyoming

**Keywords:** bacterial multicellularity, cell size, cell aspect, filament length, NtcA regulation

## Abstract

The model cyanobacterium *Anabaena* sp. PCC 7120 exhibits a phototrophic metabolism relying on oxygenic photosynthesis and a complex morphology. The organismic unit is a filament of communicated cells that may include cells specialized in different nutritional tasks, thus representing a paradigm of multicellular bacteria. In *Anabaena*, the inorganic carbon and nitrogen regime influenced not only growth, but also cell size, cell shape, and filament length, which also varied through the growth cycle. When using combined nitrogen, especially with abundant carbon, cells enlarged and elongated during active growth. When fixing N_2_, which imposed lower growth rates, shorter and smaller cells were maintained. In *Anabaena*, gene homologs to *mreB*, *mreC*, and *mreD* form an operon that was expressed at higher levels during the phase of fastest growth. In an *ntcA* mutant, *mre* transcript levels were higher than in the wild type and, consistently, cells were longer. Negative regulation by NtcA can explain that *Anabaena* cells were longer in the presence of combined nitrogen than in diazotrophic cultures, in which the levels of NtcA are higher. *mreB*, *mreC*, and *mreD* mutants could grow with combined nitrogen, but only the latter mutant could grow diazotrophically. Cells were always larger and shorter than wild-type cells, and their orientation in the filament was inverted. Consistent with increased peptidoglycan width and incorporation in the intercellular septa, filaments were longer in the mutants, suggesting a role for MreB, MreC, and MreD in the construction of septal peptidoglycan that could affect intercellular communication required for diazotrophic growth.

**IMPORTANCE** Most studies on the determination of bacterial cell morphology have been conducted in heterotrophic organisms. Here, we present a study of how the availability of inorganic nitrogen and carbon sources influence cell size and morphology in the context of a phototrophic metabolism, as found in the multicellular cyanobacterium *Anabaena*. In *Anabaena*, the expression of the MreB, MreC, and MreD proteins, which influence cell size and length, are regulated by NtcA, a transcription factor that globally coordinates cellular responses to the C-to-N balance of the cells. Moreover, MreB, MreC, and MreD also influence septal peptidoglycan construction, thus affecting filament length and, possibly, intercellular molecular exchange that is required for diazotrophic growth. Thus, here we identified new roles for Mre proteins in relation to the phototrophic and multicellular character of a cyanobacterium, *Anabaena*.

## Abstract (precis)

Most studies on the determination of bacterial cell morphology have been conducted in heterotrophic organisms. Here, we present a study of how the availability of inorganic nitrogen and carbon sources influence cell size and morphology in the context of a phototrophic metabolism, as found in the multicellular cyanobacterium *Anabaena*. In *Anabaena*, the expression of the MreB, MreC, and MreD proteins, which influence cell size and length, are regulated by NtcA, a transcription factor that globally coordinates cellular responses to the C-to-N balance of the cells. Moreover, MreB, MreC, and MreD also influence septal peptidoglycan construction, thus affecting filament length and, possibly, intercellular molecular exchange that is required for diazotrophic growth. Thus, here we identified new roles for Mre proteins in relation to the phototrophic and multicellular character of a cyanobacterium, *Anabaena*.

## INTRODUCTION

In bacteria, cell shape and cell size are key determinants of their interactions with the surrounding milieu. They present a rich diversity of cellular morphologies, which are heritable and adaptive (see references 1 and 2). Bacteria range in cell volume over at least 10 orders of magnitude (see reference 3), and cell size is also a heritable trait that can be modulated within a certain range. Recent data revealed that, at least in some model bacteria, metabolism, growth, and cell cycle progression play main roles in size setting (4).

In Escherichia coli and Bacillus subtilis, the current prevalent paradigm to explain the homeostasis of cell size is the so-called adder principle, which states that, regardless of the size at birth, a constant cell volume is added at each generation under constant growth conditions (5). Considering the dynamics of cell size variations in response to environmental conditions, at least at the population level, a relationship appears to exist between the growth rate, determined by the availability of nutrients, and cell size, so that the volume added in each generation would depend on nutrient availability. It has been proposed that the ratio between the rates of surface area growth and volume growth is a key parameter in adjusting cell size (6). However, little is known at the molecular level about how cell growth is sensed and how it could impact the initiation of cell division as a determinant of cell size (6, 7). The unicellular cyanobacterium Synechococcus elongatus has been observed to follow a sizer-like model, so that the amount of material added depends on environmental conditions and the circadian clock, which modulates growth and constricts the time window of cell division (8). In E. coli and B. subtilis, the cell size in rich medium can duplicate that in minimal medium, and an effect of carbon availability in the initiation of cell division has been described (9–11).

Regarding the determination of cell morphology, the cell wall, which allows the bacterial cell to cope with the internal osmotic pressure, has a pivotal role (12). Hence, the spatiotemporal dynamics of cell wall synthesis during the cell cycle is a key determinant of cell shape during cell growth and of its maintenance during cell division (13–15). The peptidoglycan (PG) forming the cell wall is made of long glycan strands bridged by short peptides that form a giant polymer, the murein sacculus. Considerable insight has been gained into the spatial dynamics of PG growth in bacteria that exhibit rod shape or variations thereof, such as E. coli and B. subtilis, in which PG synthesis takes place in the cylindrical part of the cell during growth. The multiprotein complex for lateral PG growth, the elongasome, integrates synthetic enzymes, including penicillin-binding proteins (PBPs), PG hydrolases, and the RodZ, MreB, MreC, and MreD proteins or homologs thereof that contribute to the peripheral localization of the PG processing enzymes (13, 15–17). MreB is an actin structural homolog characteristic of rod-shaped bacteria (18). MreB and MreC have been described as essential proteins in Caulobacter crescentus (19). In B. subtilis, MreB and MreC, but not MreD, are essential under standard growth conditions, although cells depleted of MreB or MreC can be propagated in the presence of high concentrations of magnesium (20). In E. coli, MreB, MreC, and MreD are essential (21), although cells lacking these proteins can propagate as small spheres under conditions of slow growth (22).

Much less is known about the mechanisms of determination of other bacterial cell morphologies (see references 23 and 24). Hence, toward the biological challenge of understanding how the environment influences the strain-specific shape and size of bacteria and their variations, data on different strains with disparate life modes should be accumulated.

Cyanobacteria are phototrophic organisms of ancient origin, in which the process of oxygenic photosynthesis evolved. Hence, these organisms have played a crucial role in the evolution of our planet and life on it. Currently, cyanobacteria are responsible for a large fraction of the oceans’ primary productivity at a global scale, thus significantly influencing climate dynamics (e.g., 25). The photoautotrophic mode of life has a global impact on the physiology and anatomy of cyanobacteria, and an example of this is the conspicuous presence of an intracellular membrane system, the thylakoids, where the photosynthetic apparatus is harbored (26). Regarding the assimilation of nitrogen, most cyanobacteria preferentially utilize inorganic nitrogen, and nitrate and ammonium are excellent nitrogen sources for growth. In addition, many strains are able to fix atmospheric nitrogen and, indeed, make a principal contribution to N_2_ fixation in the oceans (27). The cyanobacteria are diderm bacteria classified as Gram-negative bacteria, although their PG sacculus has several layers, which makes cyanobacteria intermediate between model Gram-negative and Gram-positive bacteria (28). Cyanobacteria show a remarkable diversity of cellular sizes and morphologies, and in addition to unicellular forms, strains characterized by the formation of different types of cell aggregates, including linear unbranched or branched filaments, are found (26, 29).

Morphology is a complex character in filamentous cyanobacteria. In genera such as *Anabaena* and *Nostoc*, the organismic unit is a uniseriate filament of cells that have been considered spherical, ovoid, ellipsoid, or cylindrical in shape (29). The cells in the filament are delimited by individual cytoplasmic membranes, and the peptidoglycan sacculus, which surrounds each cell, is thickened and frequently fussed in the intercellular septa. However, the outer membrane is continuous, thus delimiting a continuous periplasm that is shared by all the cells of the filament (30). Besides the periplasmic connection, neighboring cells in the filament are linked by proteinaceous complexes, called septal junctions, that traverse the septal PG and bridge the cytoplasm of contiguous cells, providing cell-to-cell cohesion and intercellular communication functions, as has been studied in the model strain *Anabaena* sp. strain PCC 7120 (here, *Anabaena*) (31). Moreover, depending on external factors, the filament may include different cell types specialized in different functions. Thus, when utilizing combined nitrogen, all the cells of the filament are equivalent. In contrast, under conditions of combined-nitrogen scarcity, *Anabaena* forms heterocysts, which are cells specialized in the fixation of N_2_, at semiregular intervals along the filament, resulting in a pattern of heterocysts separated by intervals of ca. 10-to-15 vegetative cells (32). Heterocysts fix N_2_ and transfer organic nitrogen-rich compounds to the vegetative cells, which in turn perform photosynthetic CO_2_ fixation and transfer organic carbon-rich compounds to the heterocysts. Hence, these organisms represent a unique case of division of labor regarding nutritional function in a pluricellular bacterium (31).

Despite their remarkable features and global significance, studies of the determination of cell morphology in filamentous cyanobacteria, as in cyanobacteria in general, are scarce (see, however, references 33 and 34). Moreover, little is known about the homeostasis of cell size in cyanobacteria, as representatives of bacteria that exhibit a metabolism relying on phototrophy and the photosynthetic assimilation of inorganic nitrogen, in contrast to the better-studied heterotrophic bacteria. We addressed here the study of morphological parameters of *Anabaena* growing under different nutritional contexts and in different regulatory-mutant backgrounds and investigated the role of the *mreB*, *mreC*, and *mreD* genes in morphology determination in this organism.

## RESULTS

We addressed the determination of morphological parameters through the different growth phases in batch cultures of *Anabaena* using inorganic combined nitrogen (either nitrate or ammonium) or N_2_ as a nitrogen source and two different regimes of inorganic carbon supply, air (low carbon, LC) and air plus a supplement of 10 mM NaHCO_3_ in the culture medium (high carbon, HC). We also studied mutants lacking a functional *ntcA* gene, which encodes a transcriptional regulator of nitrogen assimilation that is required for growth using nitrate or N_2_ (35), or lacking a functional *hetR* gene, which encodes a transcriptional regulator specifically required for diazotrophic growth (see reference 36).

### Dynamics of *Anabaena* cell growth in batch cultures.

To settle defined conditions for the study of the dynamics of cell size and morphology, the growth of *Anabaena* was followed during 28 days of incubation under each of the nutritional conditions indicated above (Fig. S1). Under these conditions, the fastest exponential growth (FEG) was observed during the first ca. 48 h in the presence of nitrate or ammonium, whereas in the absence of combined nitrogen, a lag of ca. 20 h, a period in which heterocyst differentiation takes place, preceded the exponential growth. Generally, the growth rate decreased after the first week of incubation, and by the fourth week growth ceased in cultures with ammonium or N_2_ and LC (stationary phase), whereas in the presence of nitrate, slow growth was maintained. (Table 1 presents the growth rates during each of the 4 weeks of incubation).

**TABLE 1:** Growth rate constants of *Anabaena* and mutants *ntcA*, *hetR*, *mreB*, *mreC* and *mreD*

Strain	Condition	Growth rate constant (day^−1^)^*a*^				
		Phase				
		FEG	Wk 1	Wk 2	Wk 3	Wk 4
PCC 7120 (WT)	N_2_ LC	0.35	0.262	0.149	0.065	−0.024
	N_2_ HC	0.35	0.274	0.125	0.065	0.029
	NO_3_^–^ LC	0.86	0.605	0.094	0.053	0.031
	NO_3_^–^ HC	0.54	0.466	0.077	0.072	0.055
	NH_4_^+^ LC	0.90	0.576	0.127	0.026	−0.005
CSE2 (*ntcA*)	NH_4_^+^ LC	0.497	0.449	0.139	0.022	0.012
CSSC2 (*hetR*)	NO_3_^–^ LC	0.473	0.410	0.146	0.043	0.019
	NO_3_^–^ HC	0.497	0.425	0.101	0.043	0.022
	NH_4_^+^ LC	0.466	0.427	0.122	0.007	−0.001
CSCV1 (*mreB*)	NO_3_^–^ LC	0.574	0.401	0.142	0.053	0.036
	NO_3_^–^ HC	0.480	0.365	0.108	0.053	0.029
	NH_4_^+^ LC	0.566	0.389	0.156	0.046	0.019
CSCV4 (*mreC*)	NO_3_^–^ LC	0.499	0.432	0.156	0.062	0.026
	NO_3_^–^ HC	0.370	0.226	0.190	0.091	0.043
	NH_4_^+^ LC	0.557	0.355	0.134	0.067	−0.038
CSCV2 (*mreD*)	N_2_ LC	0.156	0.110	0.18	0.139	0.046
	N_2_ HC	0.197	0.067	0.22	0.067	0.019
	NO_3_^–^ LC	0.538	0.422	0.139	0.043	0.026
	NO_3_^–^ HC	0.504	0.336	0.168	0.053	0.041
	NH_4_^+^ LC	0.581	0.473	0.127	0.041	0.012

a Growth rate constant, μ (day^−1^), corresponds to ln2/*t*_d_, where *t*_d_ is the doubling time, calculated from the increase in the optical density at 750 nm (OD_750_) in each time interval with values from 4 independent cultures of each condition (see growth curves in Fig. S1). FEG (fastest exponential growth) corresponded to growth during the first 48 h for BG11 and BG11_0_ + NH_4_^+^ media, and during 24 to 72 h for BG11_0_ medium. Mann-Whitney tests were performed to assess significance of differences with the mean values calculated from the independent experiments (Data Set S1).

10.1128/mSphere.00747-20.1 FIG S1 Growth of *Anabaena* with different nitrogen and carbon supplies. Cells grown in BG11 medium supplemented (HC) or not (LC) with 10 mM NaHCO_3_^–^, in BG11_0_ + NH_4_^+^ medium or in BG11_0_ medium (lacking combined nitrogen), supplemented or not with bicarbonate, were used to inoculate, at an initial cell density corresponding to 0.2 μg chlorophyll/ml, flasks containing the same medium, which were incubated under culture conditions. At the indicated times, the optical density at 750 nm (OD_750_) (At) was measured in aliquots of each culture. The values of 4 independent cultures of each condition were represented and adjusted to sequential linear functions. A0, OD_750_ at the start of culture. Download FIG S1, PDF file, 0.1 MB. Copyright © 2020 Velázquez-Suárez et al. 2020 Velázquez-Suárez et al. This content is distributed under the terms of the Creative Commons Attribution 4.0 International license.

10.1128/mSphere.00747-20.6 DATA SET S1 Statistical tests of data on growth rate, cell size, cell aspect, and filament length. Download Data Set S1, XLSX file, 0.1 MB. Copyright © 2020 Velázquez-Suárez et al. 2020 Velázquez-Suárez et al. This content is distributed under the terms of the Creative Commons Attribution 4.0 International license.

When incubated with LC, the FEG rate was similar with nitrate or ammonium and was approximately 0.4-fold under diazotrophic conditions (the significance of comparisons can be found in Data Set S1). Although the assimilation of nitrate, which involves nitrate transport and intracellular reduction to ammonium, is energetically more expensive than the assimilation of ammonium (35), it can be considered that under the illumination conditions used here, reducing power is not limiting for nitrate reduction, which is a process directly linked to photosynthesis (37). Regarding diazotrophic growth, the large investment of cellular resources, energy and reductants, required for heterocyst differentiation and N_2_ reduction to ammonium can explain the lower diazotrophic growth rate in comparison to growth with combined nitrogen. Fixing N_2,_ HC did not affect the FEG, although it had a positive effect on the phases of slowest growth. In the presence of nitrate, the FEG rate with HC was ca. 0.4-fold smaller than the rate observed with LC, although this difference has low statistical significance. Finally, no growth was detected in the presence of ammonium and HC.

Although both the *ntcA* and *hetR* mutants can grow with ammonium, the growth rate was ca. half that in the wild type. In the *hetR* mutant, which in contrast to the *ntcA* mutant can grow with nitrate, the growth rate was similar with LC or HC and about half that in the wild type with LC (Table 1).

### Dynamics of cell size during growth using different C and N supplies.

**FIG 1:**
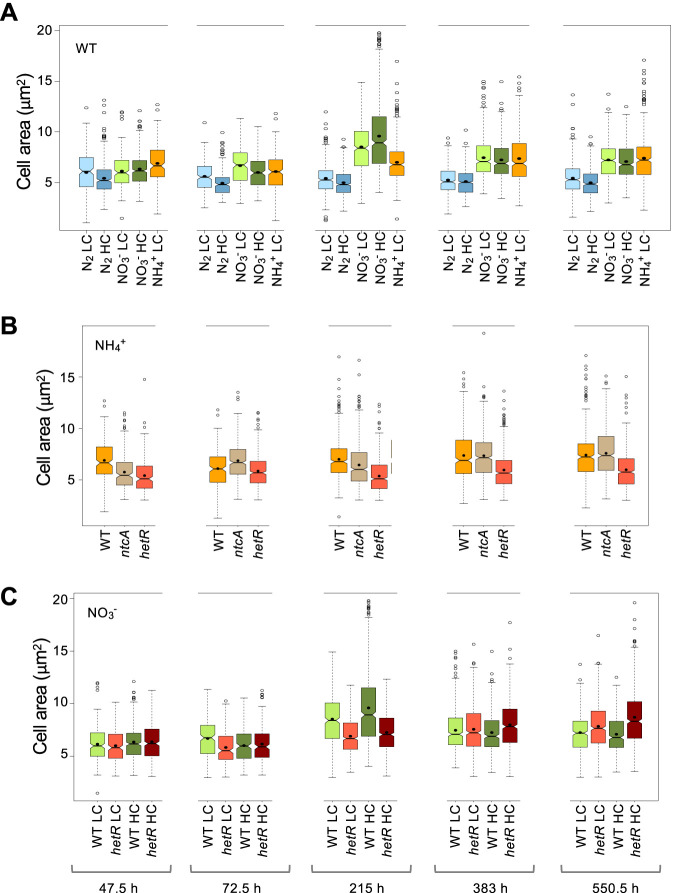
Dynamics of cell area in *Anabaena* and strains CSE2 (*ntcA* mutant) and CSSC2 (*hetR* mutant) grown with different nitrogen and carbon supplies. Cells grown in BG11 medium with low carbon (LC) or high carbon (HC), in BG11_0_ + NH_4_^+^ medium with LC, or in BG11_0_ medium (lacking combined nitrogen) with LC or HC, were used to inoculate, at an initial cell density corresponding to 0.2 μg chlorophyll/ml, flasks containing the same medium, which were incubated under culture conditions. At the indicated times, aliquots of each culture were photographed and used for cell area determination, as described in Materials and Methods. A total of 500 to 700 cells (vegetative cells in the diazotrophic cultures) from three or four different cultures of each time and condition were measured. Notched boxplot representations of the data are shown. The mean values are represented by black dots. Tukey tests were performed to assess significance of differences (Data Set S1). (A) WT; (B) *ntcA* and *hetR* mutants in BG11_0_ + NH_4_^+^; (C) *hetR* mutant in BG11.

We measured the cell area during growth under the conditions described above (Fig. 1; the significance of comparisons can be found in Data Set S1). Generally, the carbon supply had only a small impact on cell area. Cells incubated with combined nitrogen tended to increase area throughout the growth curve until reaching the stationary phase (550.5 h in Fig. 1A), so that they appeared smaller when more actively growing. Cells incubated with ammonium or nitrate were similar in size except for a transient peak that, in the transition from more active to slower growth, was repeatedly observed only with nitrate (215 h in Fig. 1A). In contrast, vegetative cells of diazotrophic cultures showed a more homogeneous cell size during growth, being smaller than cells incubated with nitrate or ammonium. Thus, diazotrophy results in not only a slower growth rate and smaller cell size than when using combined nitrogen, but also in a restriction for cell mass increase throughout the growth cycle.

To assess whether any correlation existed between the growth rate and the cell size in *Anabaena*, the growth rate constant was plotted against the mean cell area for the different nutrient conditions in each of the considered growth periods (Fig. S2). *Anabaena* showed a positive correlation during the phases of more active growth (see FEG and first week in Fig. S2), although this correlation was nonlinear. Notably, a linear negative correlation was observed with data corresponding to the 2nd week of growth, which corresponded to the transit to phases of less active growth and, in the case of nitrate-grown cells, of larger cell size.

10.1128/mSphere.00747-20.2 FIG S2 Relationships between cell size and cell growth in *Anabaena* incubated with different nitrogen and carbon supplies. Data are taken from Table 1 (growth rate constants) and Fig. 1 (mean cell area). R^2^ represents the coefficient of determination (the square of the Pearson’s coefficient of correlation). Download FIG S2, PDF file, 0.1 MB. Copyright © 2020 Velázquez-Suárez et al. 2020 Velázquez-Suárez et al. This content is distributed under the terms of the Creative Commons Attribution 4.0 International license.

Regarding the *ntcA* and *hetR* mutants, cells of the former, with ammonium and LC, were generally similar to those of the wild type (Fig. 1B). Under these conditions, cells of the *hetR* mutant were smaller than those of the wild type or the *ntcA* mutant. Also, during fast growth in the presence of nitrate, with LC or HC, cells of the *hetR* mutant were similar or somewhat smaller than those of the wild type (Fig. 1C).

### Filament length responds to the C and N nutrition.

**FIG 2:**
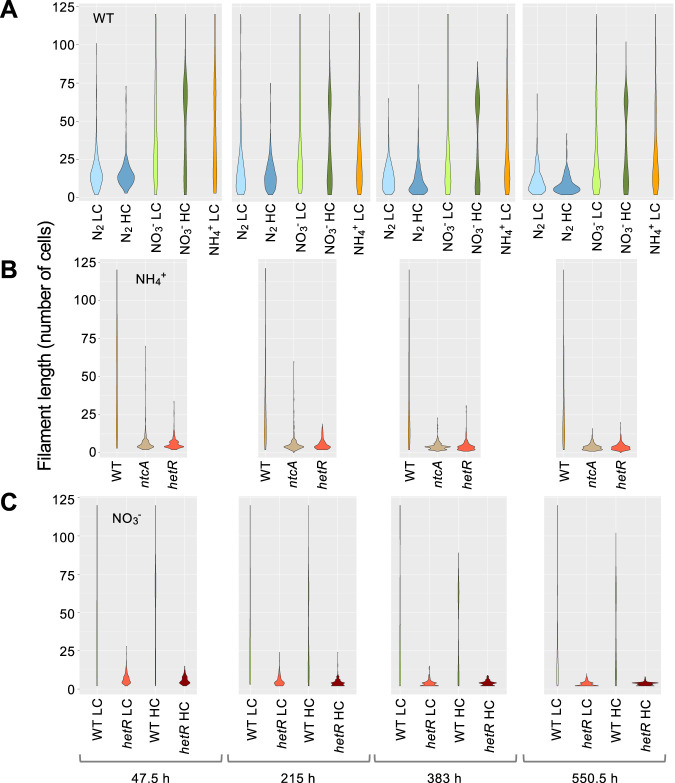
Distribution of filament length in *Anabaena* and strains CSE2 (*ntcA* mutant) and CSSC2 (*hetR* mutant) grown with different nitrogen and carbon supplies. At the indicated times, aliquots of cultures treated as described in the legend to Fig. 1 were taken with care to avoid filament breakage and photographed. Filaments from three independent cultures of each condition were counted. Filaments longer than 120 cells were counted as of 120. Violin-plot representations of the data are shown. Mann-Whitney tests were performed to assess significance of differences with data from filaments up to 119 cells long (214 to 387 filaments) (Data Set S1).

The number of cells per filament was counted through the growth cycle under the different nutritional conditions described above, and the distribution of filament lengths was analyzed (Fig. 2; the significance of comparisons can be found in Data Set S1). In the wild type, filaments tended to be longer during the phases of more active growth. Perhaps senescence and death of random intercalary cells, which would be more frequent during less active growth, could contribute to shorten the filaments.

Filaments incubated with nitrate and LC were similar in size to those incubated with ammonium and tended to be shorter than those incubated with nitrate and HC. A larger difference was observed between filaments incubated with nitrate or ammonium and the shorter diazotrophic filaments. Perhaps the septa between vegetative cells and heterocysts are more fragile than the septa between vegetative cells, contributing to shortening of filaments under diazotrophic conditions. Moreover, because heterocysts are terminally differentiated cells, senescence and lysis of old heterocysts would increase filament breakage. This is consistent with the fact that filaments of 5 to15 cells, roughly coincident with the size of vegetative-cell intervals separating two heterocysts, were the most frequently observed under diazotrophic conditions.

Finally, both in the *ntcA* mutant and in the *hetR* mutant, the filaments were much shorter than in the wild type.

### Dynamics of cell morphology during growth using different C and N supplies.

**FIG 3:**
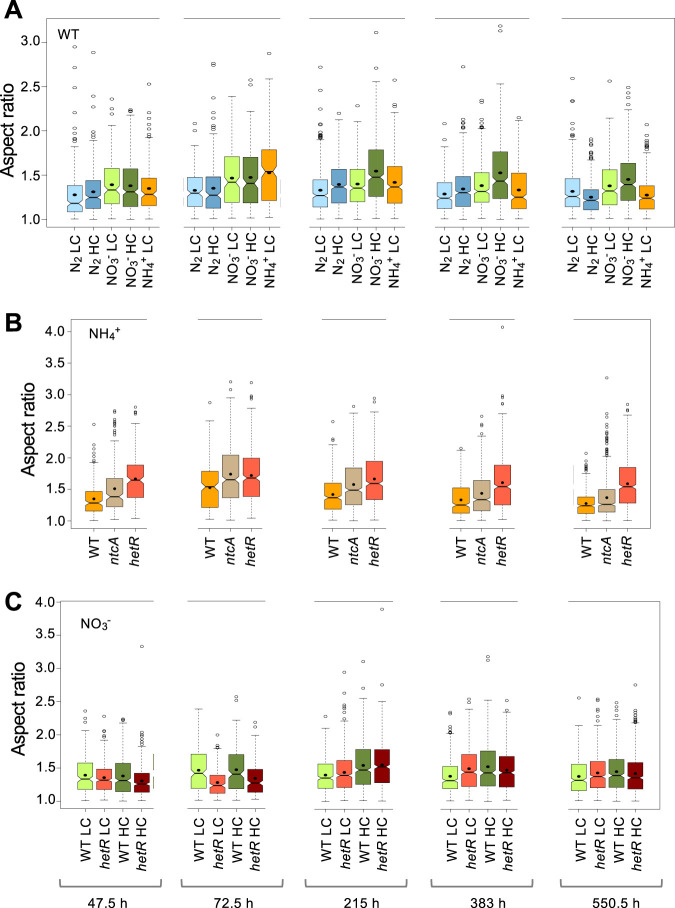
Aspect ratio of cells of *Anabaena* and strains CSE2 (*ntcA* mutant) and CSSC2 (*hetR* mutant) grown with different nitrogen and carbon supplies. In the same cells used in Fig. 1, the lengths of the longitudinal and transversal cell axes were measured as described in Materials and Methods. The aspect ratio is the result of dividing the length of the axis parallel to the filament by the length of the axis perpendicular to the filament. Notched boxplot representations of the data are shown. The mean values are represented by black dots. Tukey tests were performed to assess significance of differences (Data Set S1). (A) WT; (B) *ntcA* and *hetR* mutants in BG11_0_ + NH_4_^+^; (C) *hetR* mutant in BG11.

The lengths of the longitudinal (parallel to the filament) and the transversal (perpendicular to the filament) axes of the cells were determined in *Anabaena* under the different growth conditions used and, as an indication of cell aspect, the ratio between the two cell axes (aspect ratio) was calculated (Fig. 3; the significance of comparisons can be found in Data Set S1). In nitrate- and ammonium-supplemented medium, with LC, the aspect ratio increased through the phases of more active growth and then decreased more pronouncedly in the presence of ammonium. This same trend was observed for cells incubated under diazotrophic conditions, although in this case the aspect ratio increase was smaller and delayed, consistent with a delayed FEG (see above), compared to growth in combined nitrogen-supplemented medium.

The aspect ratio of cells incubated with nitrate or ammonium reached higher values than in diazotrophic cultures, and HC supplement generally had a positive effect. Because in rod-shaped cells an increase in length would increase the total surface area, it could have larger benefits when growth depends on nutrients taken up from the external medium (see reference 1), such as nitrate, ammonium or bicarbonate, and during phases of faster growth, and could facilitate light adsorption also. In contrast, under diazotrophic conditions, it is assumed that most N_2_ enters the heterocysts from the neighboring vegetative cells by intercellular connections (38). Moreover, because elongation is likely more costly than making short rods, the energetic balance would represent another factor favoring *Anabaena* cells being longer when combined nitrogen is available.

Notably, in ammonium-supplemented medium, the aspect ratio of the *ntcA* and *hetR* mutants was considerably higher than that of the wild type (Fig. 3B). In the *hetR* mutant, the aspect ratio was higher in cultures with ammonium than in those with nitrate (see Fig. 3B and C).

### The *mre* gene cluster of *Anabaena*.

**FIG 4:**
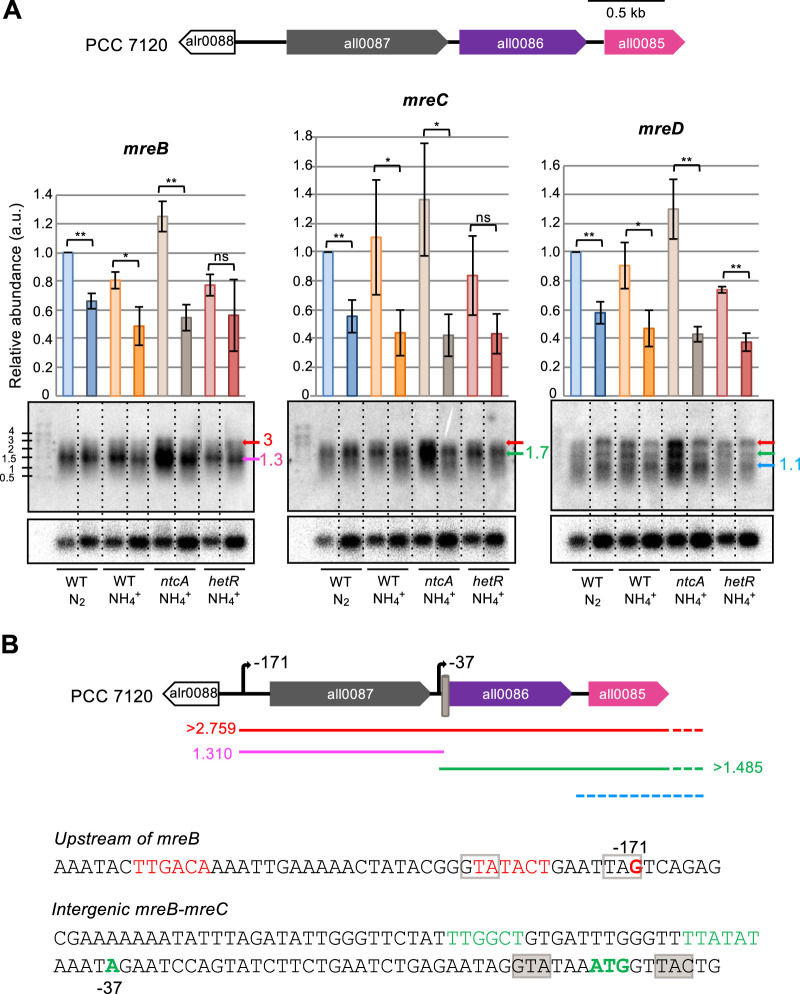
Expression of the *Anabaena mre* gene cluster. (A) Northern-blot analysis of the expression of the *mreB*, *mreC*, and *mreD* genes (genomic cluster represented in the upper part). RNA was extracted from filaments of the indicated strains grown in BG11_0_ or BG11_0_ + NH_4_^+^ medium, refreshed in the same medium at a cell density of 0.2 μg chlorophyll/ml, and incubated under culture conditions for 24 h (lighter colors) or 360 h (darker colors). Hybridization was performed with probes of the indicated *mre* gene (upper panels) or of *rnpB* used for normalization (lower panels). Three different cultures were used for each strain and condition (one representative filter of each strain and condition is shown). The mean and standard deviation of total transcript abundance, normalized for the *rnpB* signal for each lane, are represented, and significance of differences was assessed with Student’s *t* test (**, *P* < 0.01; *, *P* < 0.05; ns, *P* > 0.05). Colored arrows at the right side of each panel point to the main transcripts detected (size in kb indicated). A size standard (RiboRuler High Range, Thermo) is shown at the left. (B) Schematic model of transcription in the *mre* gene cluster. In the upper part, the localization and size of expected transcripts is depicted (dashed segments indicate unprecise localization or transcript end). TSPs located at 171 nucleotides upstream from *mreB* and 37 nucleotides upstream from *mreC* are indicated, and a gray barrel represents a putative NtcA-binding sequence. Nucleotide sequences upstream from *mreB* and in the *mreB-mreC* intergenic region are depicted. Colored sequences denote putative −10 and −35 promoter elements; colored bold, transcription start points and the ATG start of *mreC*. Shadowed GTA and TAC sequences represent a putative NtcA-binding site (consensus sequence GTAN_8_TAC), and framed GTA and TAG sequences represent an imperfect NtcA-binding site.

In the *Anabaena* genomic sequence, the *mreB* gene (all0087) and a homologue to *mreC* (all0086) are clustered together, being separated by 95 nucleotides. Downstream of *mreC*, open reading frame (ORF) all0085 is found, which is separated from the putative *mreC* by 114 nucleotides (see Fig. 4A). *Anabaena* MreB has 347 residues with 52% identity to E. coli MreB. No transmembrane helix is present in *Anabaena* MreB (checked with TMHMM) although, similar to E. coli MreB (39), it may include an amphipathic in-plane membrane anchoring segment (Amphipaseek, NPS@ [Network Protein Sequence Analysis]) for interaction with the cytoplasmic membrane. MreC is a bitopic protein with a transmembrane helix and a large periplasmic part (40). The predicted product of all0086 has 273 amino acids (aa) and 19% identity to E. coli MreC (367 aa), bears an N-terminal signal peptide locating almost the whole protein in the periplasm (Phobius), and includes a coiled-coil motif encompassing residues ca. 60 to 90 (Network Protein Sequence Analysis [41]). Finally, all0085 would encode a 170-aa product with 19% identity to E. coli MreD (162 aa). However, in spite of the low overall sequence identity, All0085 would include 5 transmembrane helices with a periplasmic N terminus and cytoplasmic C terminus, similar to the organization of E. coli MreD (Phobius, TMHMM). Thus, we will consider all0085 to be a putative *mreD* gene of *Anabaena.*

### Expression of the *Anabaena mre* gene cluster.

Expression of the *Anabaena mreBCD* cluster was studied by means of Northern blot analysis. RNA was extracted from filaments of the wild type and the *ntcA* and the *hetR* mutants in the FEG phase (24 h) and in a period of slower growth (360 h) using ammonium or N_2_ as the nitrogen source. Consistent with previous results (42), different transcripts hybridized with the probe of each gene. We found transcripts of ca. 3 and 1.3 kb (*mreB*), of ca. 3 and 1.7 kb (*mreC*), and of ca. 3, 1.7 and 1.1 kb (*mreD*) (Fig. 4A). For the different strains and conditions, the levels of *mreB* transcripts were always higher than those of *mreC*, which were higher than those of *mreD* (Table 2). For the different nitrogen sources, total transcript levels of any of the three genes were higher in the FEG phase than during slow growth (Fig. 4A; Student’s *t* test *P* values between 0.019 and 0.001, except for *mreB* and *mreC* in the *hetR* mutant, which gave *P* values higher than 0.05). No significant differences were detected comparing ammonium-grown and diazotrophic filaments (Student’s *t* test *P* > 0.05). In the presence of ammonium, total transcript levels were higher in the *ntcA* mutant than in the wild type (Student’s *t* test *P* = 0.003 for comparison of *mreB* transcript levels in the exponential phase; *P* = 0.050 for *mreD*). This result, together with the fact that the longer (ca. 3 kb) transcript was better detected in the *ntcA* mutant than in the wild type (WT), indicates a negative effect of NtcA on the expression of the *mre* genes. No significant effect of the *hetR* mutation was detected.

**TABLE 2:** Comparisons of the expression of the *Anabaena mreB*, *mreC*, and *mreD* genes by Northern blot analysis^*a*^

Strain/condition	Time (h)	Ratio of total transcript levels		
		*mreB*/*mreD*	*mreC*/*mreD*	*mreB*/*mreC*
PCC7120 (WT)/N_2_	24	4.21 ± 1.03	3.52 ± 1.43	1.28 ± 0.26
	360	3.69 ± 0.09	4.21 ± 1.79	1.11 ± 0.56
PCC 7120 (WT)/NH_4_^+^	24	4.08 ± 0.85	3.44 ± 0.83	1.29 ± 0.46
	360	4.50 ± 1.49	3.77 ± 1.31	1.29 ± 0.40
CSE2 (*ntcA*)/NH_4_^+^	24	4.91 ± 1.42	3.23 ± 0.96	1.61 ± 0.49
	360	4.85 ± 2.21	3.07 ± 0.98	1.51 ± 0.35
CSSC2 (*hetR*)/NH_4_^+^	24	5.20 ± 0.40	3.21 ± 1.01	1.76 ± 0.46
	360	5.87 ± 1.32	3.64 ± 0.48	1.59 ± 0.15

a RNA was extracted from filaments of the indicated strains grown in BG11_0_ (N_2_) or BG11_0_ + NH_4_^+^ (NH_4_^+^) medium for 24 or 360 h (see Fig. 4A for further details). The figures represent the mean and standard deviation of the ratio of total transcript levels of the corresponding gene pair in three independent cultures of each strain and condition.

**FIG 5:**
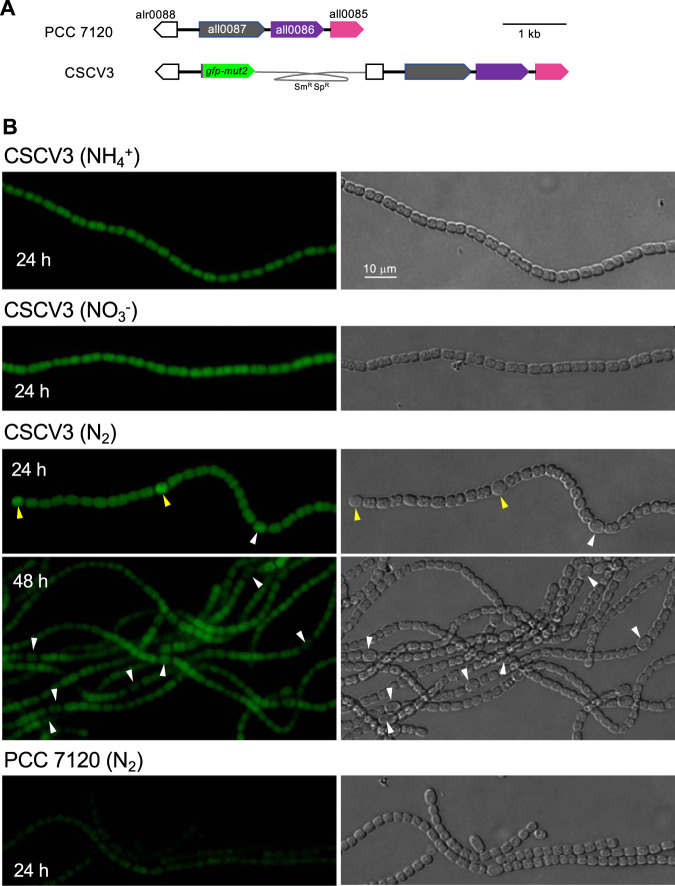
Spatiotemporal expression of the *mreB* gene promoter in *Anabaena.* (A) Genomic structure of strain CSCV3 (expressing [P*_mreB_*-*gfp*] in comparison to PCC 7120 [WT]). (B) Filaments of strains PCC 7120 and CSCV3 grown in solid BG11 medium were transferred (at a cell density of 0.2 μg chlorophyll/ml) to BG11, BG11_0_ + NH_4_^+^, and BG11_0_ media and incubated under culture conditions. At 24 h intervals, filaments were observed under a fluorescence microscope and photographed. GFP fluorescence (left) and bright-field (right) images are shown. Arrowheads point to heterocysts: immature heterocysts (yellow); mature heterocysts, exhibiting polar refringent cyanophycin granules (white). Magnification is the same for all micrographs.

We also studied the spatial pattern of the activity of the *mreB* promoter along the *Anabaena* filament. In a previous report, a *gfp* gene under the control of the *mreB* promoter introduced in *Anabaena* in a shuttle vector established with several copies per chromosome copy was reported to produce higher fluorescence in heterocysts than in vegetative cells (42). Here, we generated strain CSCV3, an *Anabaena* derivative that includes a *gfp-mut2* gene fused after the 6th codon of the *mreB* sequence, thus preserving the transcription and translation start signals of native *mreB*, in the chromosomal *mreB* locus. In addition, an intact copy of *mreB*, *mreC*, and *mreD* preceded by their native promoter region was preserved (Fig. 5A; see Materials and Methods for details).

Green fluorescent protein (GFP) fluorescence was monitored through the growth cycle in filaments of strain CSCV3 incubated with nitrate or ammonium or in the absence of combined nitrogen. In the latter case, the filaments included vegetative cells and cells in different stages of differentiation into heterocysts. Importantly, growth of strain CSCV3 was not impaired in comparison to the wild type (not shown). In cultures containing nitrate or ammonium, green fluorescence above the background in the wild-type strain was homogenously observed along the filament during active growth (Fig. 5B). Maximum fluorescence was observed during the first 24 to 48 h of incubation. After that time, fluorescence decreased, so that after 168 h, only background fluorescence was detected (Fig. S3). Upon N-stepdown, fluorescence levels increased in differentiating cells, which exhibited higher levels than vegetative cells (see 24 h in Fig. 5B). However, this increase was transitory, so that in mature heterocysts, GFP fluorescence levels were similar to those of vegetative cells (see 24 h in Fig. 5B). Indeed, 48 h after the transfer, mature heterocysts exhibited very low or undetectable GFP fluorescence, lower than that in vegetative cells. (Of note, overexpression of the P*_mreB_-gfp* reporter utilized previously [42] could have led to maintenance of increased fluorescence for longer than the expression of the promoter activity in the native context.) Meanwhile, increased fluorescence was observed in stretches of vegetative cells that were neighbors to heterocysts, likely those that were most actively growing. After 72 h, the fluorescence in vegetative cells had decreased also (not shown). Thus, activity of the *mreB* promoter is maximum during the FEG phase, and upon N-stepdown expression transiently increases in the cells differentiating into heterocysts.

10.1128/mSphere.00747-20.3 FIG S3 Spatiotemporal expression of the *mreB* gene promoter in filaments incubated with ammonium. Filaments of strain CSCV3 (P*_mreB_*-*gfp*) grown in solid BG11 medium were transferred (at a cell density of 0.2 μg chlorophyll/ml) to BG11_0_ + NH_4_^+^ medium and incubated under culture conditions. At 24-h intervals, filaments were observed under a fluorescence microscope and photographed. GFP fluorescence (left) and bright-field (right) images are shown. Magnification is the same for all micrographs. Download FIG S3, PDF file, 0.8 MB. Copyright © 2020 Velázquez-Suárez et al. 2020 Velázquez-Suárez et al. This content is distributed under the terms of the Creative Commons Attribution 4.0 International license.

### *mre* mutants of *Anabaena*.

**FIG 6:**
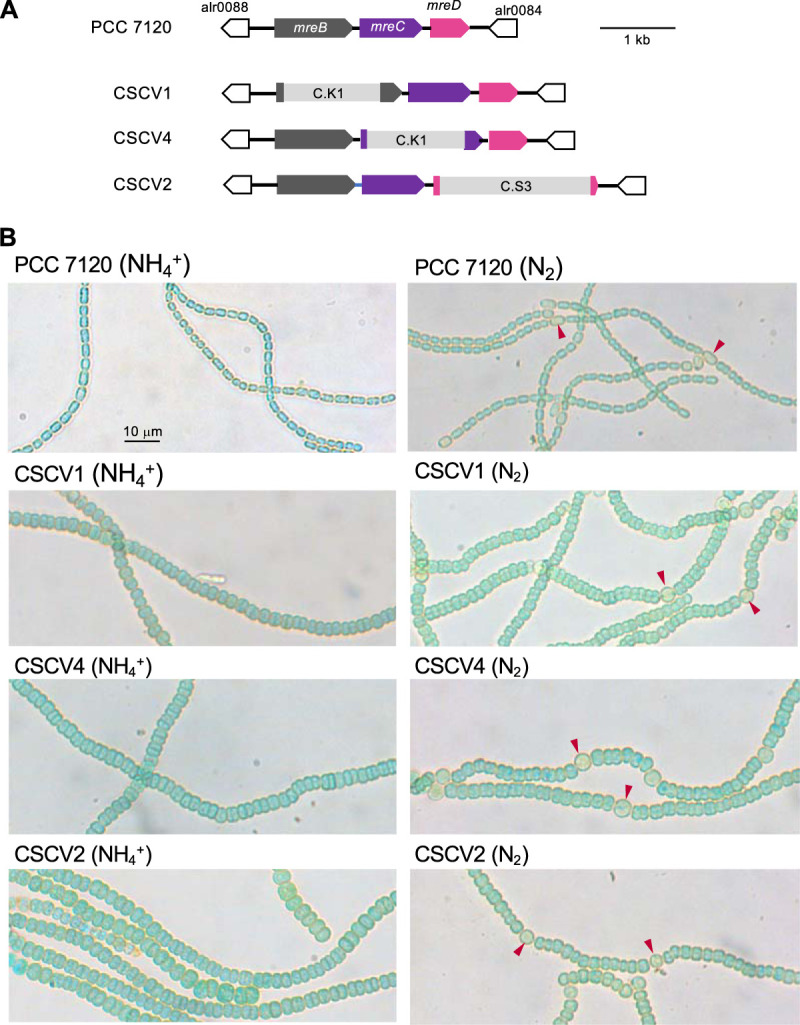
*Anabaena mreB*, *mreC*, and *mreD* mutants. (A) Genomic structure in the *mre* region in strains CSCV1 (*mreB*), CSCV4 (*mreC*), and CSCV2 (*mreD*) in comparison to *Anabaena* (WT). (B) Filaments grown in BG11_0_ + NH_4_^+^ or in BG11_0_ (lacking combined nitrogen) medium were used to inoculate, at an initial cell density corresponding to 0.2 μg chlorophyll/ml, flasks containing the same medium, which were incubated under culture conditions. After 24 h, aliquots of each culture were taken and filaments photographed. Arrowheads point to heterocysts. Magnification is the same for all micrographs.

To investigate the role of *mreB*, *mreC*, and *mreD* genes in *Anabaena*, derivatives carrying inactivated versions of these genes were created. Mutants were generated by substituting parts of each gene by an antibiotic-resistance-encoding gene cassette (Fig. 6A). Gene cassette C.S3 was inserted into the coding sequence of *mreD*. To avoid polar effects, gene cassette C.K1, which includes no transcriptional termination sequence, was inserted into genes *mreB* and *mreC* (see Materials and Methods for details). Strains CSCV1, CSCV4, and CSCV2 bear only deleted versions of *mreB*, *mreC*, and *mreD* genes, respectively (see Fig. S4).

10.1128/mSphere.00747-20.4 FIG S4 Genomic structure and segregation of mutant strains CSCV1 (*mreB*), CSCV4 (*mreC*), and CSCV2 (*mreD*). The *mre* genomic region in *Anabaena* and the mutants is schematized. C.K1 and C.S3 indicate the inserted gene cassette. PCR was performed with DNA from *Anabaena* and the mutants and the indicated primer pairs (approximate position denoted with gray arrowheads). The sizes of the expected bands are indicated in the tables. A 0.5-kb DNA ladder (gTPbio) is shown in each gel. Download FIG S4, PDF file, 1.0 MB. Copyright © 2020 Velázquez-Suárez et al. 2020 Velázquez-Suárez et al. This content is distributed under the terms of the Creative Commons Attribution 4.0 International license.

**FIG 7:**
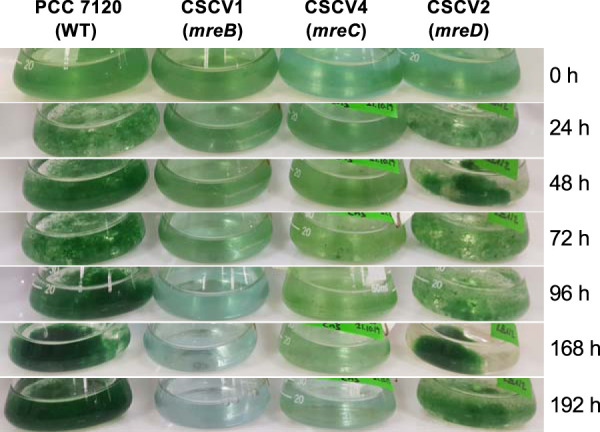
Growth of *Anabaena* and mutant strains CSCV1 (*mreB*), CSCV4 (*mreC*), and CSCV2 (*mreD*). Filaments grown in BG11_0_ + NH_4_^+^ medium were used to inoculate, at an initial cell density corresponding to 0.2 μg chlorophyll/ml, flasks containing BG11_0_ (lacking combined nitrogen) medium, which were incubated under culture conditions and photographed at the indicated times.

Strains CSCV1, CSCV4, and CSCV2 were characterized in terms of growth rate and morphology. The three mutants grew using either nitrate or ammonium, although growth was slower than in the wild type, especially with a regime of LC (the growth rate of the mutants was about 60% of that of the corresponding wild-type value) (Table 1). In the absence of combined nitrogen, the three mutants formed heterocysts (Fig. 6B). However, whereas strain CSCV2 was capable of sustained diazotrophic growth, CSCV1 and CSCV4 did not significantly increase mass and, indeed, got lysed after some days of incubation (Fig. 7). The diazotrophic growth rate of CSCV2 under LC was ca. 45% that of the wild type and 56% under HC (Table 1). To seek the cause of the impaired diazotrophic growth, nitrogenase activity was measured (Table 3). Whereas activity levels after 24 h of incubation in the absence of combined nitrogen were considerably lower in the three mutants than in the wild type, after 48 h, only strain CSCV4 was significantly impaired (activity was ca. 36% of the WT activity). Thus, the heterocysts formed in the mutants were largely active.

**TABLE 3:** Nitrogenase activity of *mreB*, *mreC*, and *mreD* mutants

Strain	Nitrogenase activity (nmol · mg clorophyll^−1^ · h^−1^)^*a*^	
	24 h	48 h
PCC 7120 (WT)	7.15	10.66
CSCV1 (*mreB*)	2.54	8.97
CSCV4 (*mreC*)	1.83	3.86
CSCV2 (*mreD*)	2.77	9.12

a Nitrogenase activity was assayed in filaments of the indicated strain incubated in BG11_0_ medium for 24 or 48 h. The figures represent the mean of the activities measured in two independent cultures that yielded similar results.

**FIG 8:**
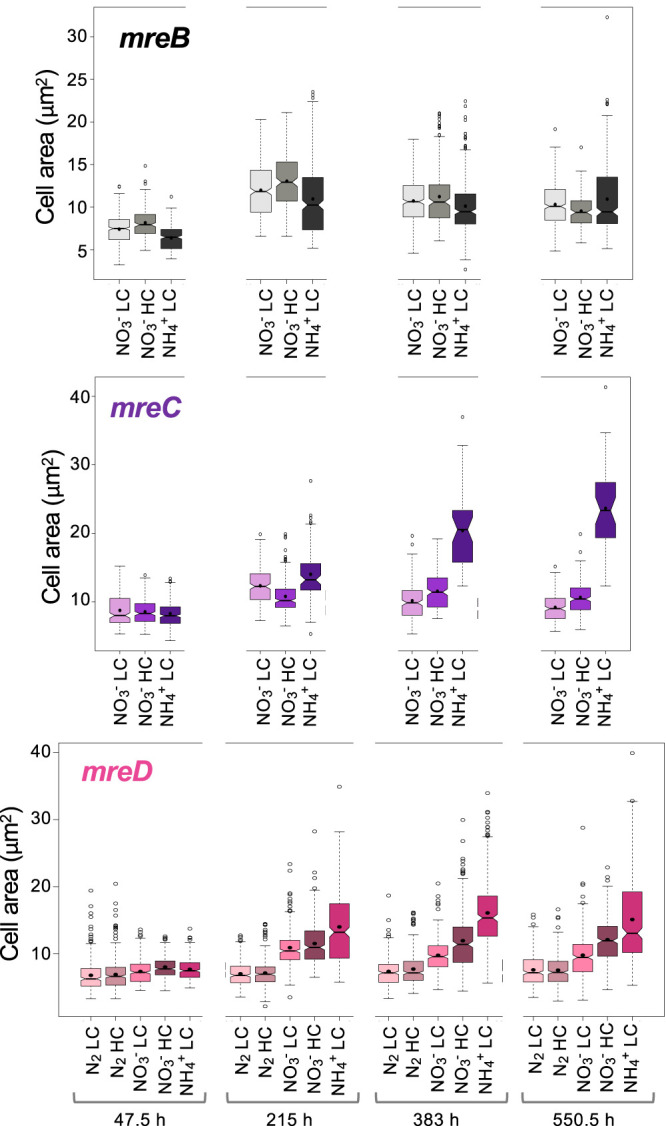
Dynamics of cell area in mutant strains CSCV1 (*mreB*), CSCV4 (*mreC*), and CSCV2 (*mreD*) grown with different nitrogen and carbon supplies. Cells grown in the indicated conditions were used to inoculate, at an initial cell density corresponding to 0.2 μg chlorophyll/ml, cultures under the same conditions. At the indicated incubation times, aliquots of each culture were photographed and used for cell area determination. A total of 200 to 400 cells (vegetative cells in the diazotrophic cultures) from three different cultures of each time and condition were measured. Notched boxplot representations of the data are shown. The mean values are represented by black dots. Tukey tests were performed to assess significance of differences (Data Set S1).

The three mutants exhibited conspicuous morphological differences compared to the wild type, in both the presence and absence of combined nitrogen (Fig. 6). Figure 8 presents data on the determination of cell area (the significance of comparisons is shown in Data Set S1). Cells of the three mutants had similar sizes in medium supplemented with nitrate (LC or HC). Notably, the mean cell area values of CSCV2 (*mreD*), and especially of CSCV4 (*mreC*), incubated with ammonium were larger than those of nitrate-incubated cells of the same strain and larger than those of ammonium-incubated cells of CSCV1 (*mreB*). Moreover, cell size appeared heterogeneous in CSCV2, showing filament stretches of larger cells and others of smaller cells (see Fig. 6), which is indicative of the inability to regulate cell size. Under any growth condition, cells of any of the three mutants (vegetative cells for filaments incubated in the absence of combined nitrogen) were larger than those of the wild type (compare Fig. 8 and Fig. 1A). As in the wild type, cells growing diazotrophically (CSCV2) were smaller than those growing with combined nitrogen (Fig. 8).

**FIG 9:**
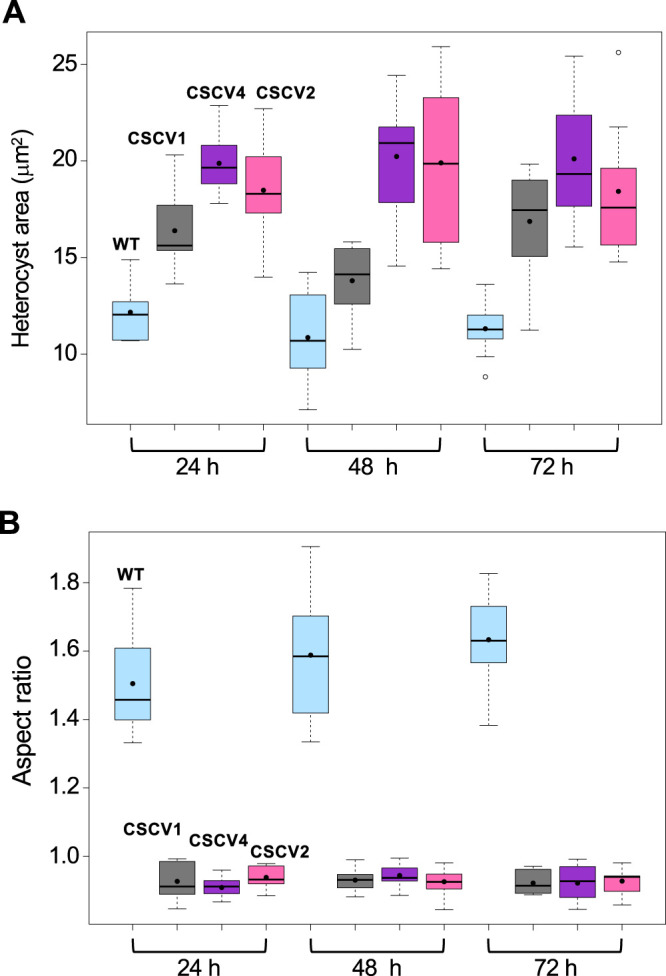
Morphological parameters of heterocysts in *Anabaena* and mutant strains CSCV1 (*mreB*), CSCV4 (*mreC*), and CSCV2 (*mreD*). Cells grown in BG11_0_ medium and LC were used to inoculate, at an initial cell density corresponding to 0.2 μg chlorophyll/ml, flasks containing the same medium, which were incubated under culture conditions. (A and B) At the indicated times, aliquots of each culture were photographed and used for heterocyst cell area (A) and cell axis length (B) determination. The aspect ratio is the result of dividing the length of the axis parallel to the filament, by the length of the axis perpendicular to the filament. Ten heterocysts of each time and strain were measured. Boxplot representations of the data are shown. Black dots represent the mean values. Tukey tests were performed to assess significance of differences (Data Set S1).

In filaments incubated in the absence of combined nitrogen, we also measured the size of heterocysts, which in the wild type were larger than vegetative cells and relatively constant during growth (compare Fig. 9A and Fig. 1A). Heterocysts of any of the *mre* mutants were larger than heterocysts of the wild type (Fig. 9A; Data Set S1).

**FIG 10:**
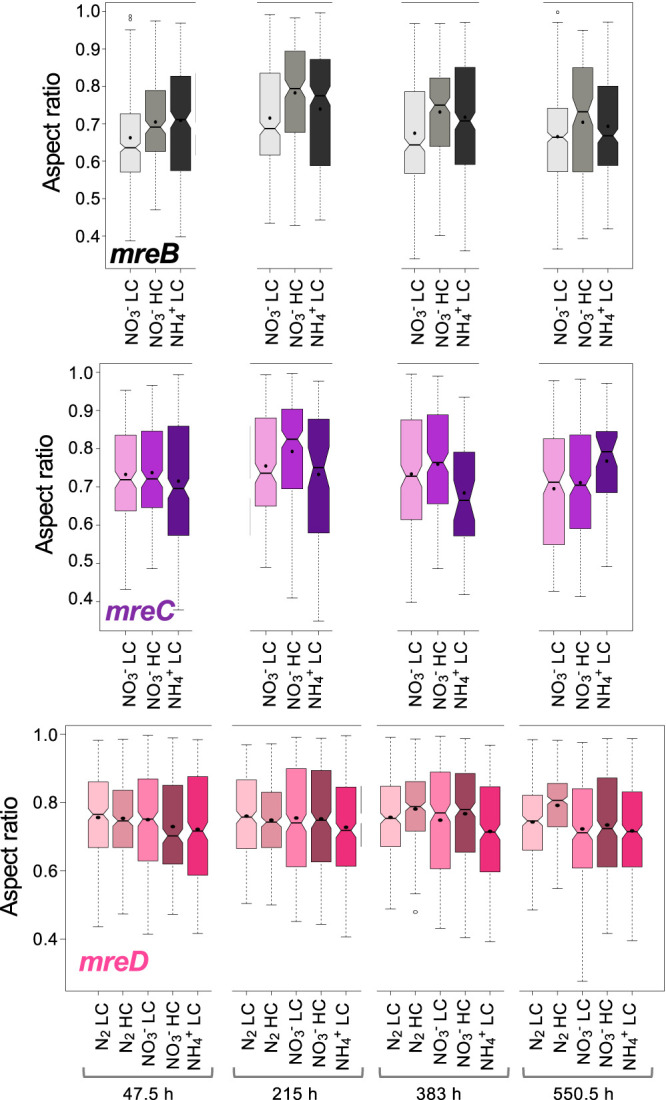
Aspect ratio of cells of *Anabaena* and mutant strains CSCV1 (*mreB*), CSCV4 (*mreC*), and CSCV2 (*mreD*) grown with different nitrogen and carbon supplies. In the same cells used in Fig. 8, the length of the longitudinal and transversal cell axes was measured. The aspect ratio is the result of dividing the length of the axis parallel to the filament, by the length of the axis perpendicular to the filament. Notched boxplot representations of the data are shown. Black dots represent the mean values. Tukey tests were performed to assess significance of differences (Data Set S1).

Regarding cell morphology, the aspect ratio of the cells of strains CSCV1, CSCV4, and CSCV2 was closer to 1 than the wild-type values (compare Fig. 10 and Fig. 3A; see Data Set S1), meaning that cells of the mutants are more rounded than cells of the wild type. Indeed, in the mutants, the aspect ratio values were smaller than 1, indicating that the cell axis parallel to the length of the filament is shorter than the axis perpendicular to the filament. Thus, the geometrical orientation of the cells in the filaments of the mutants is inverted compared to the orientation in the wild-type filaments (see Fig. 6B). Also, heterocysts of the three mutants were more rounded, and inverted, compared to the wild-type heterocysts, in which the aspect ratio was higher than in vegetative cells and showed a tendency to increase during the first 72 h of incubation (see Fig. 9B).

**FIG 11:**
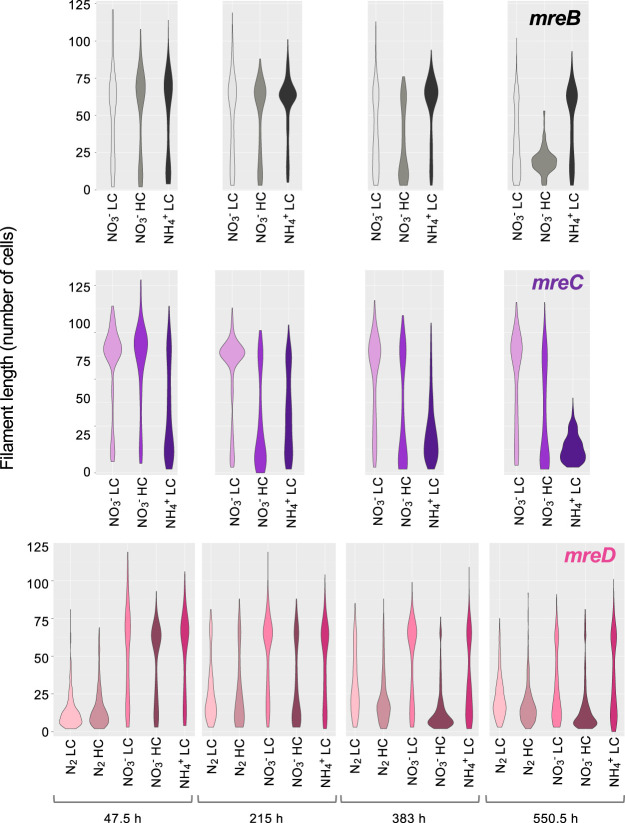
Distribution of filament length in mutant strains CSCV1 (*mreB*), CSCV4 (*mreC*), and CSCV2 (*mreD*) grown with different nitrogen and carbon supplies. At the indicated times, aliquots of cultures treated as described in the legend to Fig. 8 were taken with care to avoid filament breakage and photographed. Filaments from two to three independent cultures of each condition were counted. Filaments longer than 120 cells were counted as of 120. Violin-plot representations of the data are shown. Mann-Whitney tests were performed to assess significance of differences with data from filaments up to 119 cells long (89 to 352 filaments) (Data Set S1).

Finally, in the three *mre* mutants, the filaments were generally longer than in the wild type (compare Fig. 11 and Fig. 2A; see Data Set S1). Thus, it appears that the intercellular septa of the mutants were more robust than those of the wild type.

**FIG 12:**
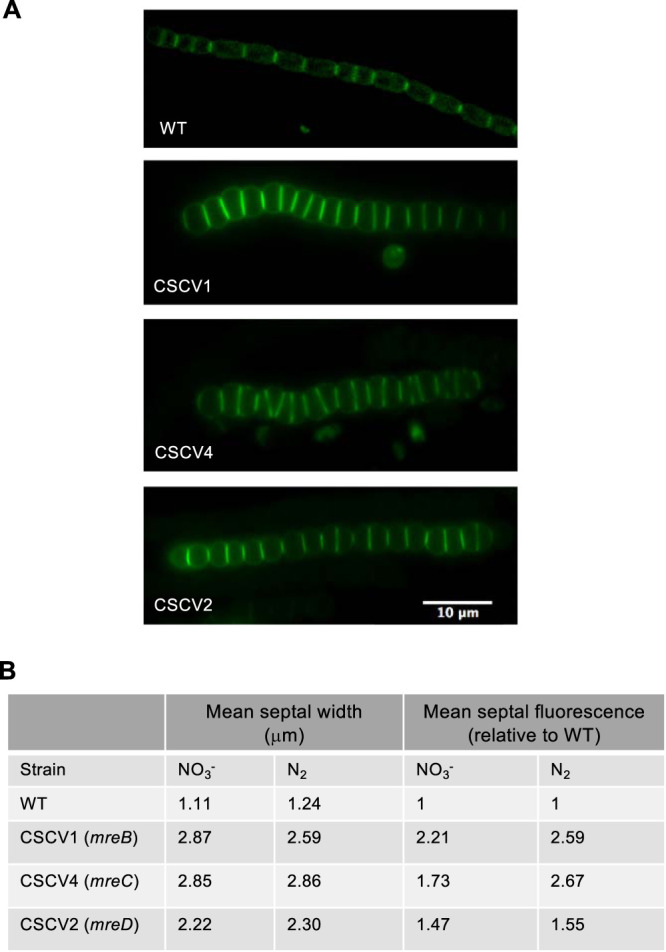
Septal width and septal Van-FL incorporation in *Anabaena* and CSCV1 (*mreB*), CSCV4 (*mreC*), and CSCV2 (*mreD*) mutants. (A) Representative fluorescence images of filaments incubated in BG11 medium and stained with Van-FL as specified in Materials and Methods. Magnification is the same for all micrographs. (B) The width of the fluorescent signal and total fluorescence detected in septa were estimated in filaments of the indicated strain incubated in BG11 (NO_3_^–^) or BG11_0_ (N_2_) medium with LC. For each strain and condition, 35 to 38 septa were measured. Student’s *t* test was used to assess significance of differences. *P* values were <0.01 for all comparisons except for the septal width of CSAV1 versus CSCV4 in BG11 (*P* = 0.9) and septal fluorescence of CSCV2 versus CSCV4 in BG11 (*P* = 0.136) and of CSCV1 versus CSCV4 in BG11_0_ (*P* = 0.748).

In order to identify possible factors contributing to the observed increased filament robustness in the *mre* mutants, we used Van-FL (fluorescent vancomycin) to estimate the septal width of vegetative cells. Van-FL labels sites of active PG synthesis (43), and in *Anabaena*, significant staining is found in the intercellular septa (e.g., 44). On average, septa were significantly wider in the mutants than in the wild type, and in CSCV1 and CSCV4, were wider than in CSCV2 (Fig. 12). Total Van-FL fluorescence detected at the septa was also higher in CSCV1 and CSCV4 than in CSCV2, which also rendered higher values than the wild type (Fig. 12). Increased septal width in the mutants can contribute to increased cell-to-cell cohesion and, thus, filament length.

## DISCUSSION

Although many previous data on the growth of *Anabaena* under various experimental conditions have been reported, here we aimed at setting defined conditions that would permit comparisons between growth rates and morphological features under different nutritional contexts. Indeed, we found that in this phototrophic bacterium, the nitrogen and carbon regime influence not only growth, but also cell size and shape and filament length.

Regarding cell size, during the FEG phase, *Anabaena* using different nitrogen sources and carbon supplies would grossly meet the classical growth law, which entails that cells of rod-shaped bacteria exhibit larger volume when they grow with a nutrient-imposed lower generation time (45), confirmed by recent studies at the population level (5). However, the positive linear correlation that was found between these two parameters in both E. coli and the slow-growing bacterium Sinorhizobium meliloti (46) could not be observed in *Anabaena* (Fig. S2). Moreover, in *Anabaena*, different relationships between growth and size were established during the growth cycle, resulting in a net cell size increase. These observations suggest that throughout active growth, the rate of cell division would progressively slow down more than the rate of total cell mass increase. In other words, the cell size at division would be increasing. Because *Anabaena* has multiple copies of the chromosome, and chromosome partitioning during cell division appears to be to a good extent aleatory (42), a large size would favor both daughter cells inheriting intact chromosomes during active growth (see reference 47).

Regarding cell shape, little has been investigated about the adaptive value of the different bacterial morphologies, but it appears that the actual bacterial shape would be the result of trade-offs between the suitability for different critical physiological tasks (see reference 2). In comparison to other studied bacteria, cell-shape transitions in *Anabaena* might be similar to the situation in the euryarchaeon Haloferax volcanii (48) and in some other bacteria, such as E. coli (49), in which a transition from rod shape (during exponential growth) to coccus (upon entry into stationary phase) has been described. However, in contrast to *Anabaena* using ammonium, in E. coli the morphological transition is concomitant with a decrease in size, which relies on continuing division without mass increase. In other bacteria, such as C. crescentus, cells adopt an elongated morphology and increased size during prolonged culture in the stationary phase (50). These contrasting responses stress the influence of the bacterial mode of life on determination of cell morphology.

Regarding the role of NtcA and HetR regulators, it is noteworthy that although both are dispensable for ammonium-dependent growth, the growth rate of the *ntcA* and *hetR* mutants was slower than that of the wild type. Moreover, during the growth cycle, cell size and aspect ratio in the mutants showed an evolution different from that in the wild type. Also, in the mutants, filaments were much shorter than in the wild type. These results abound in the tenability of HetR (and NtcA) influencing *Anabaena* physiology also in cultures supplemented with ammonium, consistent with the identification of some instances of HetR-promoted repression in the presence of ammonium (e.g., 51). The possible roles of the global regulator NtcA and of HetR in linking cell growth and cell division hint at research subjects of considerable interest.

We have studied here the spatiotemporal pattern of expression of the *Anabaena mre* gene cluster and its role in growth and morphology under different contexts. In contrast to the essential role of MreB and MreC in other studied bacteria, and in some cases of MreD also, we have found that *Anabaena* mutants lacking MreB, MreC, or MreD actively grow in the presence of nitrate or ammonium, although the growth rate was about half of that in the wild type. Our results with strain CSCV1 contrast with a previous report describing that an *Anabaena mreB* mutant was affected in cell size and morphology but unaltered in growth rate or filament length (42), although no detailed counting was provided for the latter parameter. In strains CSCV1, CSCV4, and CSCV2, extensive morphological alterations were observed. Not only were the cells less elongated than in the wild type, but also, the cell geometry with regard to the filament orientation was altered, so that the longer cell axis became perpendicular to the filament, whereas it was parallel to the filament in the wild type. In addition, in the three *Anabaena mre* mutants, especially in *mreC*, the cells were larger than in the wild type. Also, cells of the *mreD* mutant, and especially of the *mreC* mutant, were larger using ammonium than nitrate, which was not the case in the wild type. These observations suggest that in *Anabaena*, MreB, MreC, and MreD affect not only cell shape, but also the coordination between cell growth and cell division dependent on external nutrients. In addition, filaments were longer in the mutants than in the wild type, which suggests that intercellular septa are more robust in the mutants. This is consistent with the observations of increased width and apparent incorporation of septal PG in the mutants and suggests a role of Mre proteins in septal PG construction during cell division.

The variations of cell shape during the growth cycle in *Anabaena* correlate to a good extent with expression levels of the *mreB*, *mreC*, and *mreD* genes. Thus, higher expression was observed in the FEG phase, at the time that cells were increasing the aspect ratio, than during slower growth. Also, the activity of the P*_mreB_* promoter was maximal during FEG, indeed, preceding the time of maximal aspect ratio value (longer rods). Subsequently, promoter activity and gene expression levels decreased, in parallel with the decrease in the value of the aspect ratio.

Combining the results of Northern blot and P*_mreB_*-*gfp*-reporter expression analyses, we propose a transcription pattern and regulation of the *Anabaena mre* gene cluster, as depicted in Fig. 4B. The three genes can be expressed together as an operon from a promoter upstream of *mreB* (putative transcription start point [TSP] located at −171 nt [52], which is preceded by −10 and −35 promoter determinants representing a putative consensus σ^70^-type promoter). The whole operon transcript would be at least 2,759 nucleotides long, depending on its 3′ end, and would correspond to the ca. 3-kb transcript detected with the probes of the three *mre* genes. However, elongation of this transcript downstream of *mreB* could be inhibited by binding of NtcA to an NtcA-binding site (consensus sequence GTAN_8_TAC [53]), which is found overlapping the ATG start of *mreC*. Transcription halting at this point would produce a ca. 1.3-kb monocistronic *mreB* gene transcript. The observation that not only the ca. 3-kb transcript, but also the ca. 1.3-kb transcript was more abundant in the *ntcA* mutant than in the WT suggests that NtcA also represses transcription from the operon promoter (e.g., by binding to the imperfect site found overlapping the operon TSP). Additionally, joint transcripts of *mreC* and *mreD* (longer than 1,485 kb; possibly the ca. 1.7-kb transcript detected with the *mreC* and *mreD* probes) could be produced from an intergenic promoter (putative TSP located at 37 nucleotides upstream from *mreC* [53]), which could be partially blocked by NtcA also. Finally, the ca. 1.1-kb transcript detected with the *mreD* probe could correspond to a degradation product of the ca. 1.7-kb transcript. In summary, both the transcription pattern of *mreB*, *mreC*, and *mreD* genes and transcript abundance appear to be regulated by NtcA, which responds to the C-to-N balance of the cells influenced by the nitrogen regime. The negative effect of NtcA on transcript abundance is manifest when comparing the wild type and the *ntcA* mutant (Fig. 4A). This regulation could explain the higher aspect ratio observed in the *ntcA* mutant in comparison to the wild type. Also, although at the times studied here no significant differences in *mre* expression were found, the aspect ratio was lower in diazotrophic cultures, when the NtcA levels are higher (54) than in ammonium-supplemented cultures.

In contrast to growth with combined nitrogen, MreB and MreC, but not MreD, appear to be essential for diazotrophic growth of *Anabaena*. Under these conditions, vegetative cells of strain CSCV2 (*mreD*) were larger, less elongated, and inversely oriented compared to cells in wild-type filaments. In spite of the lack of growth of strains CSCV1 (*mreB*) and CSCV4 (*mreC*), the three *mre* mutants were able to form heterocysts that, although larger and altered in shape compared to the wild type, appeared similar in the three mutants. Also, the three mutants exhibited considerable levels of nitrogenase activity, although activity development was retarded compared to the wild type. (It should be noted that, although in CSCV4, nitrogenase activity was about one-third of the wild-type level, in our experience this activity suffices to maintain diazotrophic-growth rates comparable to those of wild-type *Anabaena*.) Moreover, besides CSCV2, at least CSCV1 also bore in the heterocysts cyanophycin polar granules, a polymer of Asp and Arg made after the incorporation of the ammonium resulting from N_2_ fixation into amino acids (55) (see Fig. 6). Thus, it appears that the incapacity for diazotrophic growth in the absence of MreB or MreC is not due to altered regulation of differentiation or of synthetic metabolism of the heterocysts. Moreover, the phenotype of strains CSCV1 and CSCV4 incubated in the absence of combined nitrogen is not that of a progressive yellowing, largely maintaining viability, characteristic of *Anabaena* when subjected to nitrogen deficiency. Instead, both strains differentiated heterocysts and then lysed, apparently when they started to resume growth (see Fig. 7). This progression could be the consequence of a defective lateral PG wall unable to maintain cell pressure during diazotrophic growth. In *Anabaena*, different PBPs (44, 56, 57) and cell wall hydrolyzing AmiC-type amidases (58, 59) have been described as required specifically for diazotrophic growth but not for growth with combined nitrogen. Perhaps, MreB and/or MreC are needed for regulation of enzymes specifically required for PG remodeling in the vegetative cells during diazotrophic growth. In addition, altered molecular transfer between cells in the filament, which is more stringently required under diazotrophic conditions than when combined nitrogen is available, could contribute to the lack of diazotrophic growth in *mreB* and *mreC* mutants. In this regard, a miss-dimensioned septal PG, as observed in the mutants, could interfere with the construction of septal nanopores, which after septum closure during cell division are drilled in the central part of septal PG caps (60) to hold septal junction protein complexes (see reference 31). The specific roles of Mre proteins in septal peptidoglycan construction in the filament of *Anabaena* are issues worthy of future investigation.

In summary, in *Anabaena*, a filamentous phototrophic bacterium, the cell size, filament length, and cell shape are regulated in response to the nitrogen and carbon nutrition and the growth phase, but here, the relationships between growth rate and size only partially follow those established in bacteria with other life styles. *Anabaena* bear homologs to the genes *mreB*, *mreC*, and *mreD*, whose expression is regulated by the growth phase and the transcriptional regulator NtcA, which responds to the C-to-N balance of the cells. As in other rod-shaped bacteria, Mre proteins are required for cell shape determination, but here, they are also required for the regulation of cell size and filament geometry. In addition, in *Anabaena*, at least MreB and MreC are required for the formation of active diazotrophic filaments, which might entail a role in the construction of functional intercellular septa allowing intercellular communication functions that are at the basis of the multicellular character of this bacterium.

## MATERIALS AND METHODS

### Strains and growth conditions.

*Anabaena* sp. PCC 7120 and mutant strains were grown in BG11 medium (containing NaNO_3_ as a nitrogen source), BG11_0_ (lacking combined nitrogen) (29), or BG11_0_ supplemented with 4 mM NH_4_Cl and 8 mM TES-NaOH buffer (pH 7.5). For high-carbon (HC) conditions, BG11 and BG11_0_ media were supplemented with 10 mM NaHCO_3_. Cultures were incubated at 30°C with illumination (12 μE m^−2^ s^−1^ from LED lamps) in Erlenmeyer flasks with shaking or in plates in medium solidified with 1% Difco agar. For the mutants, media were supplemented with antibiotics as follows: spectinomycin (Sp) and streptomycin (Sm) at 5 μg ml^−1^ each in solid media or 2 μg ml^−1^ each in liquid media (CSE2, CSCV3, CSCV2) or with neomycin (Nm) at 25 μg ml^−1^ in solid media or 5 μg ml^−1^ in liquid media (CSCV1, CSCV4). Strain CSE2 is an *ntcA* mutant (61); strain CSSC2 is a *hetR* mutant (62). The chlorophyll content (Chl) of the cultures was determined after extraction with methanol (63). (In *Anabaena*, 1 μg chlorophyll corresponds to ca. 3.3 × 10^6^ cells.)

Strain CSCV3 expresses a fusion of the *mreB* gene promoter to the gene encoding *gfp-mut2.* To generate it, *Anabaena* genomic DNA (isolated as described in reference 64) was used to amplify (with the oligodeoxynucleotide primers alr0088-3/all0087-9; all oligodeoxynucleotide primers are described in Table S1) a ClaI/EcoRV-ended fragment encompassing sequences upstream and the six N-terminal codons of *mreB*. This DNA fragment was cloned upstream and in-frame to the *gfp-mut2* gene in ClaI/EcoRV-digested mobilizable plasmid pCSEL22 (65), generating plasmid pCSCV7, which was transferred to *Anabaena* by conjugation (66) with selection for Sm/Sp (resistance to Sm and Sp is encoded in the vector portion of pCSEL22). One of the clones that had inserted pCSCV7 into the *Anabaena mreB* locus by a single recombination event, keeping an intact version of the gene preceded by its native promoter (analyzed by PCR, not shown), was selected and named strain CSCV3.

10.1128/mSphere.00747-20.5 TABLE S1 Oligodeoxynucleotide primes used in this work. Download Table S1, PDF file, 0.1 MB. Copyright © 2020 Velázquez-Suárez et al. 2020 Velázquez-Suárez et al. This content is distributed under the terms of the Creative Commons Attribution 4.0 International license.

Mutant strain CSCV1 carries a version of the *mreB* gene in which codons 14 to 267 were substituted by gene cassette C.K1, encoding Km/Nm resistance. To generate it, two DNA fragments were amplified from *Anabaena* genomic DNA using the primer pairs all0086-2/all0087-6 (encompassing sequences internal and upstream of *mreB*) and all0087-1/alr0088-2 (encompassing sequences internal and downstream of *mreB*), including terminal restriction sites XbaI/BglII and BglII/XbaI, respectively. Both fragments were joined together by overlapping PCR, and the resulting single fragment was cloned into mobilizable vector pCSRO (encoding the gene *sacB* for positive selection [67]). Gene cassette C.K1 was then inserted into the internal BglII site, generating plasmid pCSCV2, which was transferred to *Anabaena* by conjugation.

Mutant strain CSCV2 carries a version of the *mreD* gene in which codons 4 to 163 were substituted by gene cassette C.S3, encoding Sm/Sp resistance. To generate it, a strategy similar to that used for strain CSCV1 described above was used, with oligonucleotide pairs alr0084-2/all0085-6 (encompassing sequences internal and upstream of *mreD*) and all0085-7/all0086-5 (encompassing sequences internal and downstream of *mreD*), including terminal restriction sites XbaI/BamHI and BamHI/XbaI, respectively. The resulting fragment was cloned into plasmid vector pRL271 (encoding the gene *sacB* and a chloramphenicol resistance determinant) (68), and gene cassette C.S3 was inserted into the internal BamHI site rendering plasmid pCSCV6, which was transferred to *Anabaena* by conjugation.

Mutant strain CSCV4 carries a version of the *mreC* gene in which codons 12 to 266 were substituted by gene cassette C.K1. To generate it, two DNA fragments were generated using *Anabaena* genomic DNA and primer pairs all0085-1/all0086-11 (encompassing sequences internal and upstream of *mreC*) and all0086-12-1/all0087-7 (encompassing sequences internal and downstream of *mreC*), including terminal restriction sites XbaI/EcoRV and EcoRV/XbaI, respectively. Both fragments were joined together by overlapping PCR, and the resulting single fragment was cloned into mobilizable vector pCSRO. Gene cassette C.K1 was then inserted into the internal EcoRV site generating plasmid pCSCV4, which was transferred to *Anabaena* by conjugation.

The presence of the mutagenic gene construct was analyzed by PCR in selected clones resistant to Nm or Sm/Sp and to sucrose (sensitivity to sucrose is encoded in the vector portion of the transferred plasmids), implying that the corresponding inactivating gene construct was inserted in the *mre* locus by double recombination substituting for the corresponding native allele. The presence of chromosomes with the wild-type copy of each gene was also tested (Fig. S4). No wild-type allele was detectable in any case. One clone of each construct was selected and named strain CSCV1 (*mreB*), strain CSCV4 (*mreC*), and strain CSCV2 (*mreD*), respectively.

### Analysis of *mreBCD* expression by Northern blotting.

Total RNA from *Anabaena* was isolated as described in reference 69, and trace DNA in the samples was removed by treatment with Turbo RNase (Ambion) following the manufacturer’s instructions. Northern blot assays were performed as described in reference 70, with 4 μg RNA loaded per lane, and electrophoresed in denaturing 1% agarose formaldehyde gels. DNA probes were internal gene fragments generated by PCR using *Anabaena* genomic DNA and primer pairs all0087-22/all0087-23 (*mreB*), all0086-13/all0086-14 (*mreC*), and all0085-8/all0085-9 (*mreD*). The *rpnB* gene, which was used for normalization, was amplified from plasmid pT7-7120 (71) with the primers Universal and Reverse. Probes were labeled by annealing the PCR-generated fragments to oligonucleotides complementary to the coding strand (all0087-8/all0087-23 for *mreB*, all0086-6/all0086-14 for *mreC*, and all0085-5/all0085-9 for *mreD*) and polymerization catalyzed by the Klenow fragment of DNA polymerase (Thermo Fisher) in the presence of [α-^32^P]dCTP (Perking-Elmer). Radioactive areas in Northern blot hybridization membranes were visualized and quantified with a Cyclone storage phosphor system (Packard).

### Nitrogenase activity determination.

Nitrogenase activity was estimated by the acetylene reduction assay carried out under oxic conditions (72) in nitrate-grown filaments incubated in BG11_0_ medium (at 1 μg chlorophyll/ml, without antibiotics) for 24 or 48 h.

### Microscopy.

Cell area and cell axis length were determined automatically by processing light-microscopy images with ImageJ software (make images binary/fill holes/watershed/analyze) (https://imagej.nih.gov/ij/index.html). Data were plotted using the open source software RStudio Desktop. For Van-FL staining, filaments incubated for 24 h in solid medium were suspended in liquid medium supplemented with 2 μg/ml Vancomycin-FL (Bodipy-FL conjugate; Invitrogen) and incubated for 1 h in the dark with shaking. Filaments were washed twice with liquid medium and spotted in agar. GFP and Van-FL fluorescence was visualized with a Leica DM6000B fluorescence microscope, and FITCL5 filter (excitation band-pass, 480/40; emission band-pass, 527/30), and photographed with an ORCA-ER camera (Hamamatsu). Septum width and total septal fluorescence were determined with ImageJ processing of fluorescence images of filaments stained with Van-FL, by collecting fluorescence signals in manually defined septal sections.
