# Supplementary figures and images for: The Inorganic Nutrient Regime and the mre Genes Regulate Cell and Filament Size and Morphology in the Phototrophic Multicellular Bacterium Anabaena
Source: mSphere. 2020 Oct 28;5(5):e00747-20. doi: 10.1128/mSphere.00747-20 (PMC7593598; doi:10.1128/mSphere.00747-20)

Velázquez-Suárez *et al.*, Fig. S1

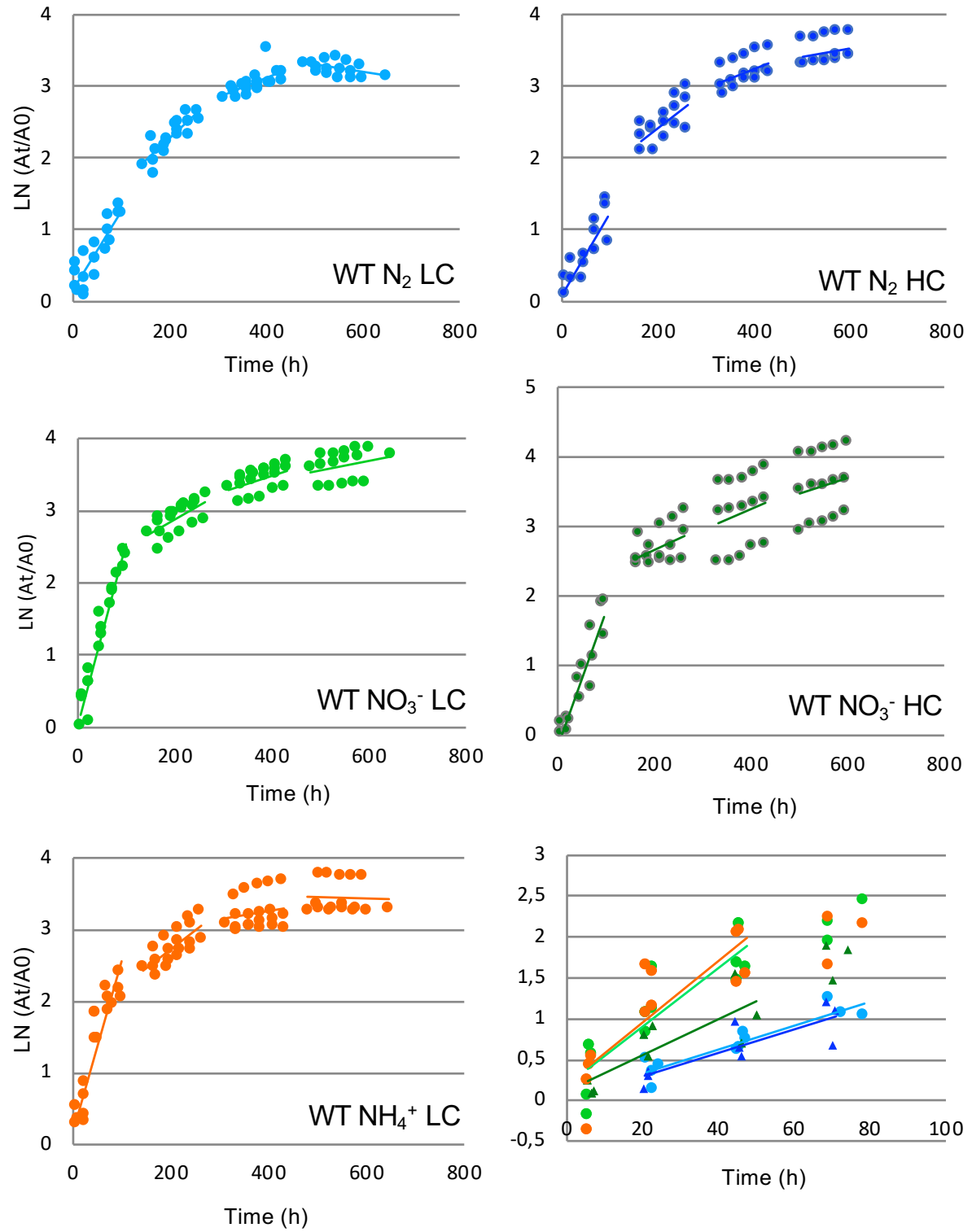

Supplement: FIG S1 [file mSphere.00747-20-sf001.pdf]

Velázquez-Suárez *et al.*, Fig. S2

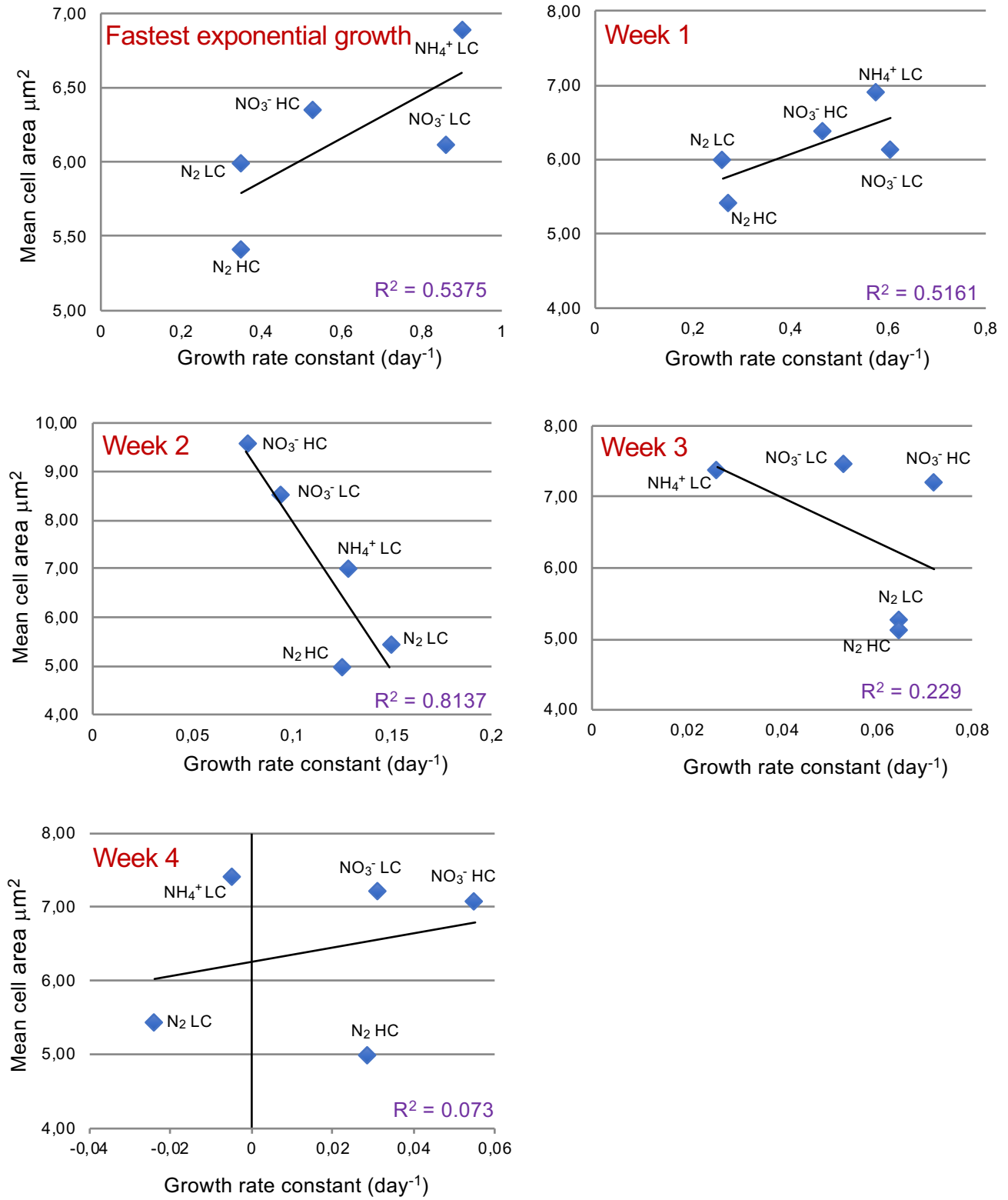

Supplement: FIG S2 [file mSphere.00747-20-sf002.pdf]

Velázquez-Suárez *et al.*, Fig. S3

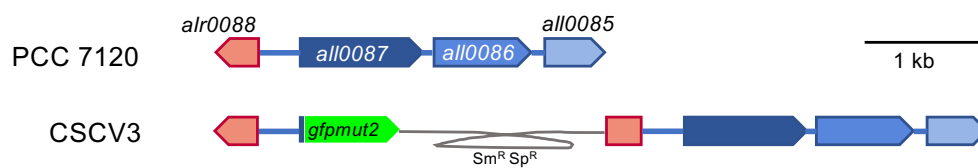

CSCV3 (NH<sub>4</sub><sup>+</sup>)

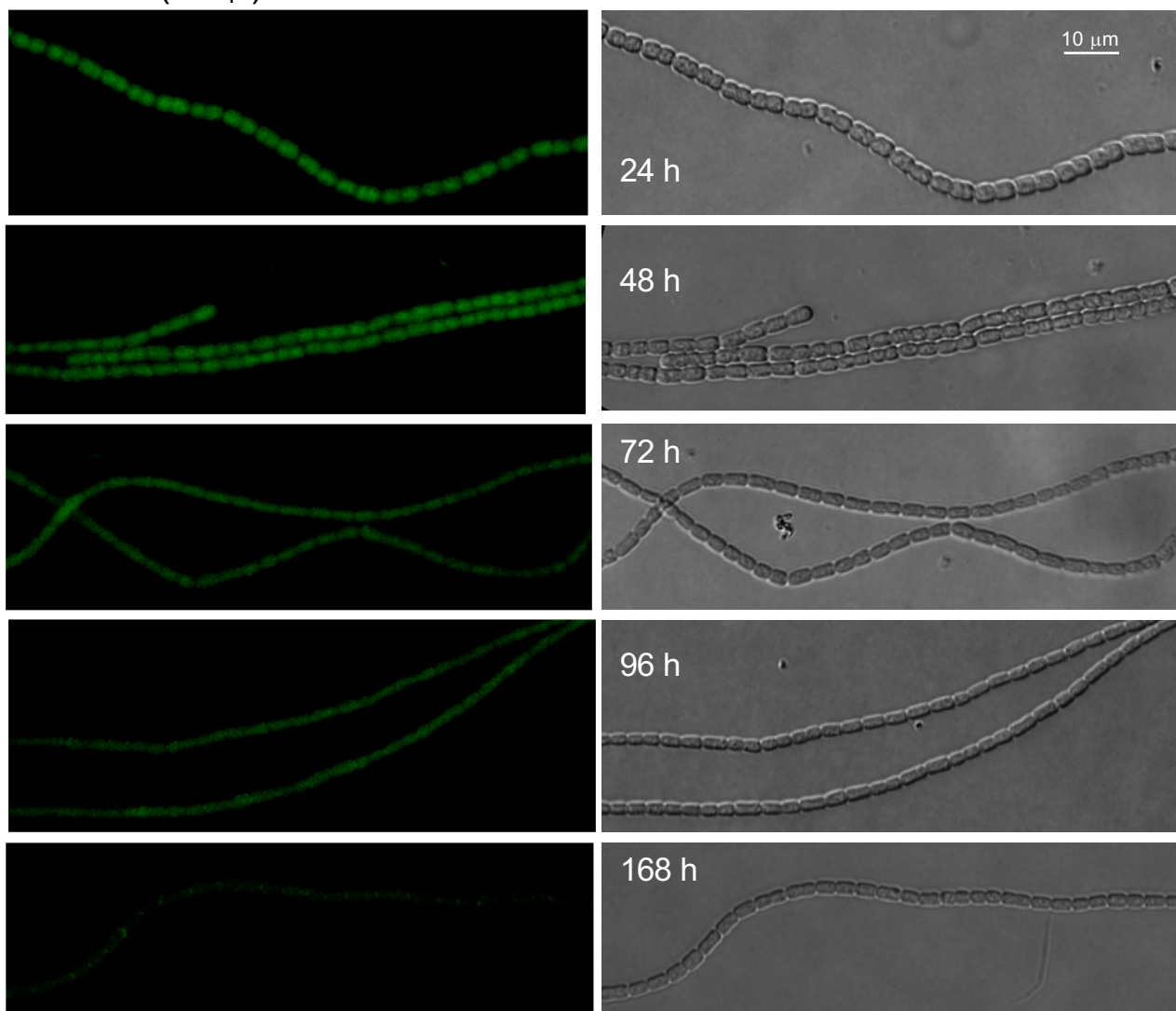

Supplement: FIG S3 [file mSphere.00747-20-sf003.pdf]

Velázquez-Suárez *et al.*, Fig. S4

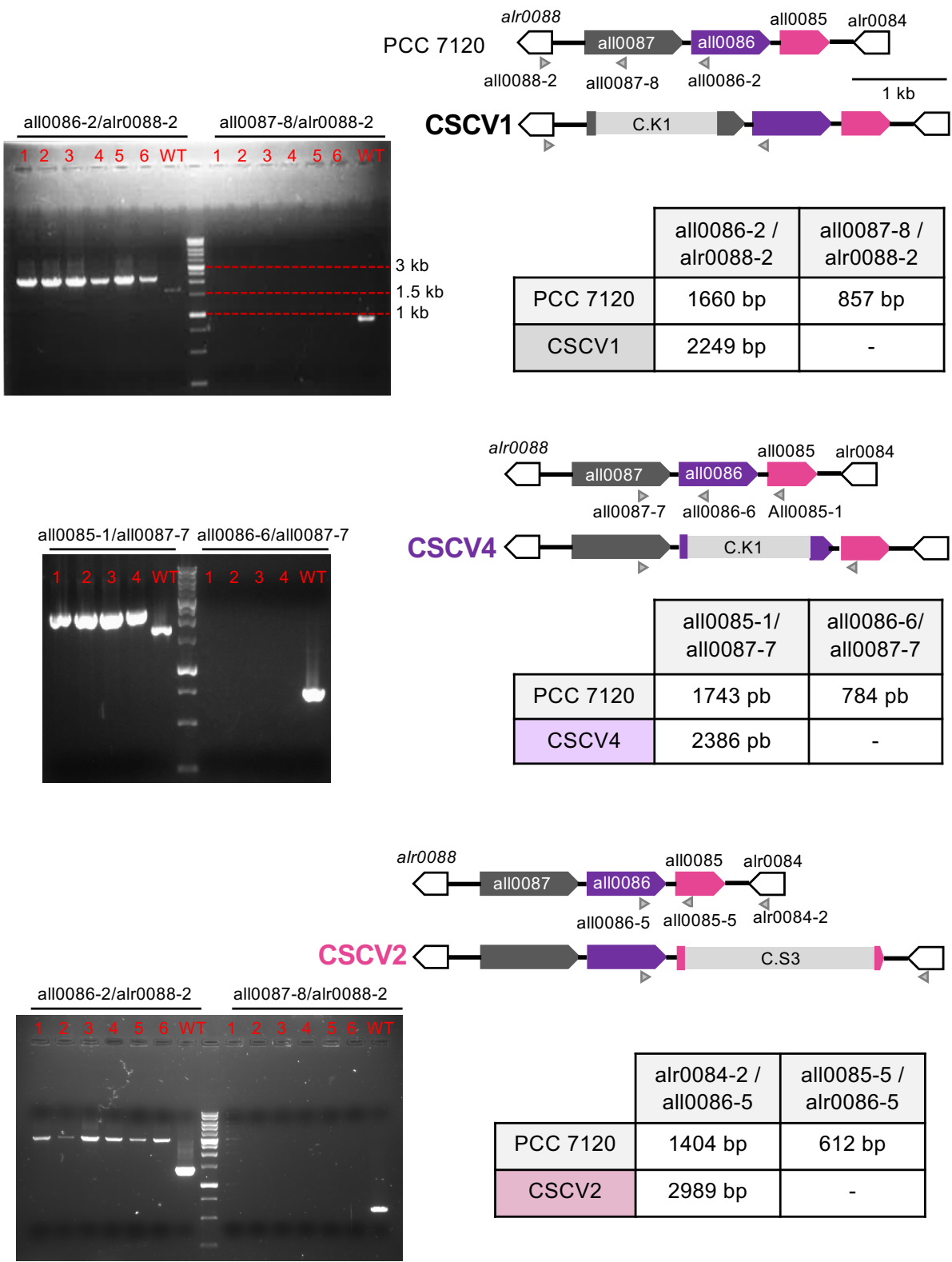

Supplement: FIG S4 [file mSphere.00747-20-sf004.pdf]
