# Supplementary material for: The Inorganic Nutrient Regime and the mre Genes Regulate Cell and Filament Size and Morphology in the Phototrophic Multicellular Bacterium Anabaena
Source: mSphere. 2020 Oct 28;5(5):e00747-20. doi: 10.1128/mSphere.00747-20 (PMC7593598; doi:10.1128/mSphere.00747-20)
Supplement: TABLE S1 [file mSphere.00747-20-st001.pdf]

TABLE S1. Oligodeoxynucleotide primers used in this work*

Name	Sequence (5’-3’)
alr0084-2	TCTAGAGGTTACGTTGTGGCTGGG
all0085-1	ACAGACTCTAGACCGTTTCTGCCAAAATTGC
all0085-5	GCAGCCCTAACACAATACCAG
all0085-6	ACTGGATCCGTGGATCCGAATGAAGATG
all0085-7	TTCGGATCCACGGATCCAGTAGGTGAAG
all0085-8	GGTGGAGGTGGAGGTATGCCTTTGTCGCGTTGG
all0085-9	GAGCTGGATCCCTAACTTTCCAACATCTTC
all0086-2	GGTATGTCTAGACTAAAAACCCGCCTTGAGT
all0086-5	CTTGAGTCTAGAGTGTTCTCCGGGGAGATT
all0086-6	GCACTGCGTCCTATCACCCTTG
all0086-11	GTCCGATATCTCACAGCAACAAAAGTC
all0086-12	CTTGATATCCTTTGCGATCCCACCAAC
all0086-13	GGTGGAGGTGGAGGTATGGTTACTGTACGTCGTTG
all0086-14	TTCCGGATCCTCTAGTTGGACTTTTGTTGC
all0087-1	GAGGTCAGATCTCATATCCCACGATGAGCGA
all0087-6	ATCATCAGATCTGTGAAGCGGACATTAGAAC
all0087-7	CTGGCATCTAGAGGTTTACACCTGCTCTCA
all0087-8	GTTCCGCAACTGGTAATCCCGC
all0087-9	ATTGATATCGTTCCTAAAAAGCCCCAC
all0087-18	GAGACATATGGGGCTTTTTAGGAAC
all0087-19	CAGATTCTCGAGATACTGGATTC
all0087-20	TCTGATGCATCCTTTAGTACATAAG
all0087-21	CTTTGCTCATGCGTCTCTATGCCCCCTATT
all0087-22	GGTGGAGGTGGAGGTGTGGGGCTTTTTAGGAAC
all0087-23	ATTTGGATCCCTACATATTTCGAGATCGTC
alr0088-2	CTCAAATCTAGAAATGTCTGGATCAGTGCCTAC
alr0088-3	GTAATCGATGCTACTACCACCGTCTC
all4723-3	TAGTATGCATGAGGAAGCTGGCGGTGG
all4723-4	ATAAATGCATTGTAGTTGGGGCTTGC
THRS2-1F	TTTATTCATATGGTCAGTTCCTTAAC
THRS2-1R	CGTATATTCAACTCGAGCAATCTGC
gfp-4	CAAGAATTGGGACAACTCC
SF-GFP-F	ACGTAGATCTATGAGCAAAGGAGAAGAACTTTTC
SF-GFP-R	ACCTCCACCTCCACCTTTGTAGAGCTCATCCATGCC
SF-GFP-F2	ATAGAGACGCATGAGCAAAGGAGAAGAACT
SF-GFP-R2	ACGTATGCATTTATTTGTAGAGCTCATCCATG
pRL277-1	TCAAGGATCTGGATTTCGAT
pRL277-2	AGAAGTGGTCCTGCAACTTTAT
sacB-1	CTTGAGGTACAGCGAAGTG
sacB-2	TCTGCAAAAGGCCTGGAGG
T7 promoter	TAATACGACTCACTATAGGG
T7 terminator	GCTAGTTATTGCTCAGCGG

*The underlined letters indicate a restriction site.
